# Selective Brain Activations and Connectivities Related to the Storage and Recall of Human Object‐Location, Reward‐Location, and Word‐Pair Episodic Memories

**DOI:** 10.1002/hbm.70056

**Published:** 2024-10-22

**Authors:** Edmund T. Rolls, Ruohan Zhang, Gustavo Deco, Deniz Vatansever, Jianfeng Feng

**Affiliations:** ^1^ Department of Computer Science University of Warwick Coventry UK; ^2^ Institute of Science and Technology for Brain Inspired Intelligence, Fudan University Shanghai China; ^3^ Oxford Centre for Computational Neuroscience Oxford UK; ^4^ Center for Brain and Cognition, Computational Neuroscience Group, Department of Information and Communication Technologies Universitat Pompeu Fabra Barcelona Spain; ^5^ Brain and Cognition, Pompeu Fabra University Barcelona Spain; ^6^ Institució Catalana de la Recerca i Estudis Avançats (ICREA), Universitat Pompeu Fabra Barcelona Spain

**Keywords:** episodic memory, hippocampus, memory recall, memory storage, orbitofrontal cortex, parahippocampal scene area, ventromedial visual cortical scene stream

## Abstract

Different cortical systems to the hippocampus were activated using fMRI during different types of episodic memory task. For object with scene location episodic memory, the activations were high in cortical systems involved in spatial processing, including the ventromedial visual and medial parahippocampal system. These activations for the medial parahippocampal system were higher in the right hemisphere. The activations in the face and object processing ventrolateral visual cortical stream regions FFC, PIT, V8 and TE2p were higher in the object‐location in scene task than the reward‐location task, and were higher in the right hemisphere. For reward‐location in scene episodic memory, activations were also high in the ventromedial visual cortical spatial stream to the hippocampus, but were also selectively high in storage in key reward cortical regions (ventromedial prefrontal 10r, 10v, 10d; pregenual anterior cingulate d32, p24, p32, s32; and medial orbitofrontal cortex reward‐related pOFC, 11l, OFC). For word‐pair episodic memory, activations were lower in the ventromedial visual and medial parahippocampal spatial cortical stream, and were higher in language‐related regions in Broca's area (44, 45, 47l), and were higher in the left hemisphere for these regions and for the many highly connected inferior frontal gyrus regions in the left hemisphere. Further, effective connectivity analyses during the episodic memory tasks showed that the direction of connectivity for these systems was from early visual cortical regions V2–V4 to the ventromedial visual cortical regions VMV1–3 and VVC for spatial scene processing; was from the pregenual anterior cingulate and orbitofrontal cortex reward systems to the hippocampal system; and was from the FFC/V8/PIT system to TE2p in the visual inferior temporal visual cortex, which has connectivity to lateral parahippocampal TF, which in turn has forward effective connectivity to the hippocampus.

## Graphical abstract

What, Where, and Reward systems leading to the hippocampal memory system for object‐location, reward‐location, and word pair associate episodic memory. The ventromedial “Where” cortical pathway, the ventrolateral “What” cortical pathway, and the orbitofrontal cortex and anterior cingulate cortex Reward system were selectively activated, and were connected to the hippocampus.

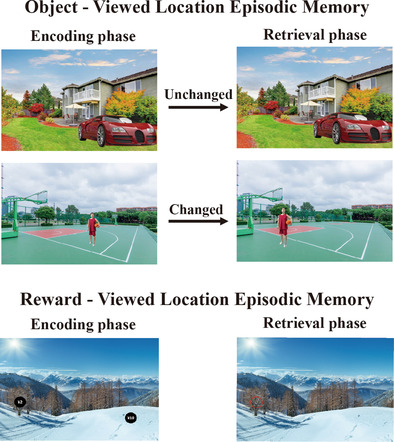

**Summary**

- In an object‐viewed location episodic memory task, the ventromedial visual cortical “Where” scene stream via the parahippocampal gyrus, and a ventrolateral object/face visual cortical “What” stream via FFC were activated and connected with the hippocampal episodic memory system.
- In a reward‐viewed location episodic memory task, the ventromedial visual cortical “Where” scene stream via the parahippocampal gyrus, and an orbitofrontal/anterior cingulate cortex reward stream were activated and connected with the hippocampal episodic memory system.
- In a word paired associate episodic memory task, the semantic system in the left anterior temporal lobe and the system in Broca's area (44, 45, and 47 L) and the adjoining inferior frontal gyrus regions were activated and connected with the hippocampal episodic memory system.

## 1 Introduction

The aim of this research is to investigate the neural storage and recall of three types of episodic memory in humans, object‐location memory, reward‐location memory, and word‐pair memory. This was performed by using fMRI to measure selective brain activations and connectivities in these three types of episodic memory tasks, during both the storage and recall of these types of episodic memory. This investigation provides new evidence on which cortical regions, and which cortical pathways connecting these regions, are involved in different types of human episodic memory, and of left–right differences in the regions activated in these three types of episodic memory. A highlight of the research is that it is performed with the Human Connectome Project MultiModal Parcellation atlas (HCP‐MMP) (Glasser, Coalson et al. 2016) enabling the cortical activations found in human episodic memory to be related to the effective connectivity maps between the 360 cortical regions in this atlas that have been obtained previously (Rolls et al. 2022b, 2023a, 2023d, 2023c; Rolls, Deco, Zhang, et al. 2023; Rolls, Feng, et al. 2023; Rolls, Wirth, et al. 2023; Rolls, Yan, et al. 2024). This adds function to connectivity maps to help better understand the functional architecture of the human cerebral cortex.

There is increasing interest in the functioning of the human hippocampus in memory, given that the representations in the human and primate hippocampus, and hippocampal connectivity, is very different in humans and other primates from rodents (Rolls 2023d, 2023c, 2024c; Rolls and Treves 2024). For example, in primates including humans, the spatial representations are especially of locations in spatial scenes being viewed as exemplified by hippocampal and parahippocampal spatial view cells (Rolls, Robertson, and Georges‐François 1997; Robertson, Rolls, and Georges‐François 1998; Rolls et al. 1998; Georges‐François, Rolls, and Robertson 1999; Ekstrom et al. 2003; Rolls and Xiang 2005, 2006; Rolls, Xiang, and Franco 2005; Ison, Quian Quiroga, and Fried 2015; Wirth et al. 2017; Rolls and Wirth 2018; Qasim et al. 2019; Tsitsiklis et al. 2020; Mao et al. 2021; Qasim, Fried, and Jacobs 2021; Donoghue et al. 2023; Rolls 2023b, 2023c; Yang, Chen, and Naya 2023; Piza et al. 2024), whereas in rodents the representations are especially of the place where the rodent is located as exemplified by hippocampal place cells (O'Keefe 1979; Burgess, Recce, and O'Keefe 1994; Hartley et al. 2014; Moser, Moser, and McNaughton 2017). In line with this difference, there is now evidence for a ventromedial visual cortical stream to the human hippocampus (Rolls 2023c; Rolls et al. 2023a; Rolls, Deco, Zhang, et al. 2023; Tullo et al. 2023; Rolls, Yan, et al. 2024) via the parahipocampal place area (Epstein and Julian 2013; Epstein and Baker 2019). A key computational implication is that spatial view representations are built in the primate including human hippocampus by ventral stream‐like visual feature combination processes to represent spatial features in scenes being viewed (Rolls 2023c, 2024c, 2024a, 2024b; Rolls and Treves 2024; Rolls and Turova 2024; Rolls, Zhang, and Feng 2024). These concepts on the spatial representations in humans and other primates have important implications for navigation (Rolls 2020, 2021) as well as for human episodic memory, with spatial view, object, and reward representations in the hippocampus important in episodic memory (Rolls and Treves 2024). Indeed, human episodic memory related to the hippocampal system (Squire and Wixted 2011; Bennett and Stark 2016; Moscovitch et al. 2016; Chrastil et al. 2018; Reznik et al. 2023) includes associations between objects/people and their spatial locations in scenes, rewards; and their spatial locations in scenes (building on evidence from neuronal recordings in macaques [Rolls and Xiang 2005; Rolls, Xiang, and Franco 2005; Rolls and Xiang 2006]), and associations between word pairs (Clark, Kim, and Maguire 2018; Glikmann‐Johnston et al. 2020; Rolls and Treves 2024).

In previous research on this ventromedial visual cortical scene stream to the human hippocampus using the Human Connectome Project Multimodal Parcellation (HCP‐MMP) atlas (Glasser, Coalson et al. 2016) described below, part of the evidence comes from measuring effective connectivity in Human Connectome Project (HCP) resting state fMRI data (Rolls et al. 2023a). Another part of the evidence comes from measuring effective connectivity in HCP magnetoencephalography data of humans viewing faces or tools, but not scenes (Rolls, Deco, Zhang, et al. 2023). This has now being extended to effective connectivity measured with magnetoencephalography when humans are viewing scenes (Rolls, Yan, et al. 2024). Function was added to these analyses by measuring activations and functional connectivity in humans viewing faces, places, tools, and body parts while performing 0‐back and 2‐back short‐term memory tasks (Rolls, Yan, et al. 2024). However, in none of these studies was an episodic memory task being used, and the aim of the research described here was accordingly to measure activation and effective connectivity for all cortical regions in the HCP‐MMP atlas during the storage and recall of episodic memory. The task used was of episodic memory in that first the humans were shown 8 images across 80 s, then counted backwards for 10 s, and then during recall had to remember whether the object was in the same scene location, or whether the reward was in the same scene location, or whether the word pairs were the same, as during the storage trials. These are tests of hippocampus‐dependent episodic memory in that tasks involving remembering multiple object‐location, reward‐location, and arbitrary associations between words are impaired by hippocampal damage (Buckley 2005; Squire and Wixted 2011; Kesner and Rolls 2015; Moscovitch et al. 2016; Clark, Kim, and Maguire 2018; Glikmann‐Johnston et al. 2020; Rolls and Treves 2024). This new design also allowed activations and effective connectivities during storage and recall to be compared, and for human brain systems involved in episodic memories for object/person and scene location, reward and scene location, and word pair associations, to be measured in the same set of participants using fMRI and the HCP‐MMP atlas. By comparing laterality, we were able to strengthen the evidence for partially separate cortical pathways all leading to the hippocampus for these three types of episodic memory. This investigation was thus able to provide new evidence on the different pathways involved in different types of human episodic memory.

A highlight of the research is the use of the HCP‐MMP1 (Glasser, Coalson, et al. 2016) which is a well‐founded parcellation of the human cerebral cortex into 360 cortical regions that utilizes evidence from anatomy (cortical thickness and cortical myelin), functional connectivity, and task‐related fMRI (Glasser, Coalson, et al. 2016). This atlas provides a reference system that could be used in many investigations of human cortical function, to provide a reference standard to enable findings from different investigations to be compared. The HCP‐MMP1 (Glasser, Coalson, et al. 2016) has been extended to include 66 subcortical areas (Huang et al. 2022). The HCP‐MMP1 is the best cortical atlas we know for delineating the smallest functional cortical regions that can be reliably identified in humans, which may be building blocks of cortical function and provide a basis for advancing our understanding of cortical function (Rolls 2023d). All the data analyzed were in the surface‐based version of the HCP‐MMP1 atlas, as that provides the most accurate identification of each cortical region (Glasser, Coalson, et al. 2016). It contrasts with many earlier parcellations of the cerebral cortex that are less computationally useful as they are based on gross topology (Rolls, Joliot, and Tzourio‐Mazoyer 2015; Rolls et al. 2020), or on cortical regions categorized primarily by functional connectivity (Power et al. 2011).

Maps of cortical connectivity have been generated for many cortical systems using this HCP‐MMP1 atlas using effective connectivity, functional connectivity, and diffusion tractography. Effective connectivity measures the connectivity in each direction between each pair of brain regions by using time delays (Rolls et al. 2022b), and was complemented by measurement of functional connectivity, which given that it is based on Pearson correlations, can provide evidence about interactions between brain regions, but not about the direction or causality of effects (Ma et al. 2022; Rolls et al. 2022c). These methods were complemented by diffusion tractography, which can measure direct connections between brain regions though not about the direction of connections (Huang et al. 2021; Rolls et al. 2022c). These three types of connectivity maps for the human cerebral cortex have been generated for the visual cortical regions (Rolls et al. 2023a; Rolls, Deco, Zhang, et al. 2023; Rolls 2024c); the posterior parietal cortex (Rolls et al. 2023c); the orbitofrontal cortex, anterior cingulate cortex, and ventromedial prefrontal cortex (Rolls et al. 2022c); the posterior cingulate and medial parietal cortex (Rolls, Wirth, et al. 2023); the auditory cortex (Rolls, Rauschecker, et al. 2023); the amygdala compared to the orbitofrontal cortex (Rolls et al. 2023b); the prefrontal and somatosensory cortex (Rolls et al. 2023d); the frontal pole cortex (Rolls et al. 2024a); and the hippocampal memory system (Huang et al. 2021; Ma et al. 2022; Rolls et al. 2022b; Rolls, Yan, et al. 2024).

## 2 Methods

### 2.1 The Episodic Memory Tasks

Three episodic memory tasks were performed by all participants during the fMRI data acquisition. The tasks were object‐location episodic memory, reward‐location episodic memory, and word‐pair association episodic memory. For each run of a task, there was an encoding or storage phase in which eight images were shown over 80 s. This was followed by a 10 s period of counting backwards. Then in the recall phase, images were shown again, and the participants had to indicate whether the object was in the same location in the scene as during encoding for the object‐location task; whether the location was the high‐reward or low‐reward location in the scene in the reward‐location task; and whether the word‐pairs were the same as during encoding for the word‐pair memory task. These are episodic memory tasks in that each image is shown only once with many images to be learned before testing occurs. Each task was repeated for three runs interleaved with the other tasks. In each 10 s trial, an image was presented for the first 5 s, and the screen was blank for the next 5 s. Details follow.

#### 2.1.1 Object‐Location Memory Task

**FIGURE 1:**
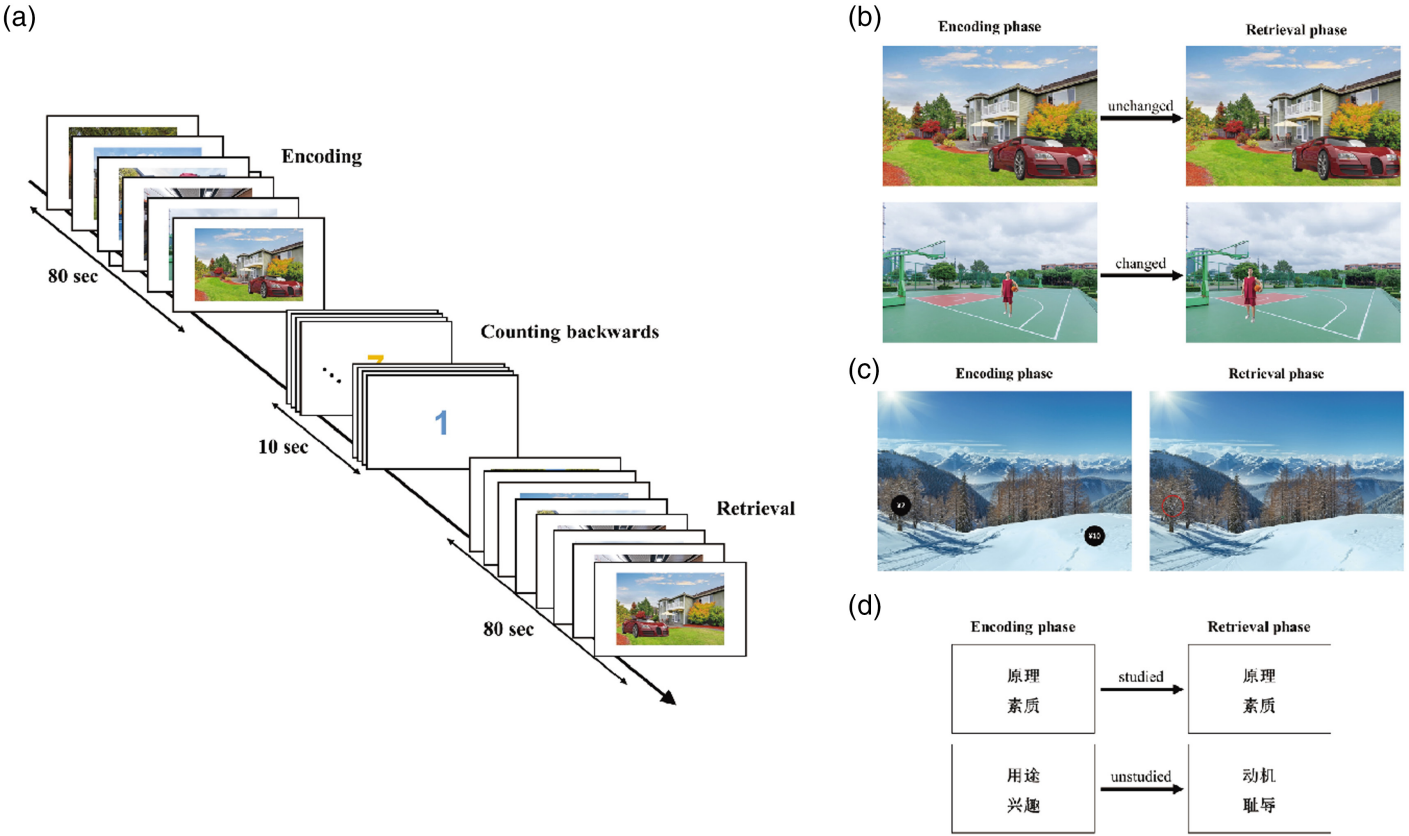
The Episodic Memory tasks. (a) The experimental paradigm of one run of the episodic memory tasks, which consists of an encoding (or storage) block, a baseline block with counting backwards, and a retrieval block. Each encoding block and each retrieval block comprise eight trials of 10 s for each trial, with an image shown in the first 5 s, and a blank screen in the second 5 s of each trial. Participants perform three runs of each of an object‐location episodic memory task, a reward = location episodic memory task, and a word‐pair episodic memory task. (b) In the object‐location memory task during each trial of the encoding block, participants are required to remember the location of the object (a person or a car) in an image of a scene. In the following baseline block, participants count backwards from 10 to 1 with the change of numbers shown on the screen. Then, during each trial of the retrieval block, participants make a button press to indicate whether the location of the object in the scene has changed compared with that shown in the encoding block. Examples of the images in the object‐location memory task are shown in the panels. The upper panel shows that the location of the car in the picture presented in the retrieval phase is unchanged compared with that presented in the storage phase, while the lower panel shows that the location of the person in the scene has changed. (c) In the reward‐location memory task during each trial of the encoding block, participants are asked to remember the locations of the big (¥10) and small (¥2) rewards marked in the scenes of the picture. Then, participants count backwards from 10 to 1 as the numbers change on the screen in the baseline block. Next, during each trial of the retrieval block, participants chose a button press to indicate whether the location marked with a red circle represents a big or a small reward in the encoding block. The left picture is an example picture presented in the encoding block, showing a scene with two locations marked with a big reward and a small reward, respectively. The right picture shows the same scene as the left one, but with a red circle, which is an example image presented in the retrieval block. (d) In the word pair associates memory task, during each trial of the encoding block, participants learned a word pair that consists of two Chinese double‐character abstract nouns. Participants then count backwards from 10 to 1 in the following baseline block. During each trial of the retrieval block, participants press a button to indicate whether the word pair shown on the screen is that shown in the encoding block. The top panel shows an example of a studied word pair in the retrieval phase that is repeated from the encoding phase. The middle panel shows an example of a recombined word pair in the retrieval phase that is a random new associate constructed from the words that were shown in the encoding phase. The bottom panel shows an example of a new word pair that had not been shown before. The recombined word pair and the new word pair are both regarded as incorrect word pairs.

To assess object‐location association episodic memory, an object‐location episodic memory task was designed based on the paradigms described by Glikmann‐Johnston et al. (2020), Bellgowan et al. (2009), and Buffalo, Bellgowan, and Martin (2006). The task employs a block design fMRI protocol, to analyze the engagement of the hippocampus in object‐location association memory. In the task, full‐length portraits of Chinese people and cars were used as the object stimuli, for these objects are easy to recognize and the participants had a clear aim to remember the location of a person or a car in each scene. The scenes were all landscapes and the object stimuli were placed naturally in the different scenes as illustrated in Figure 1b.

The task comprised three runs with three types of block: encoding, baseline, and retrieval. Each run includes an encoding or memory storage block, a 10 s counting backwards baseline block, and a memory retrieval or recall block in turn. To begin the task, participants are instructed to fixate their gaze on a cross at the center of the screen for 10 s. Following the fixation period, participants proceed to an encoding block consisting of eight trials lasting a total of 80 s, with each trial lasting 10 s. Each trial begins with an image shown for 5 s, followed by a blank screen for 5 s as the interstimulus interval. Participants are required to remember the object (a person or a car) and its location in a image of a scene, and to press a right key on the MRI response box with each stimulus presentation during a trial.

The encoding block is followed by a baseline block. In a baseline block, participants are required to fix their eyes on the numbers shown in the center of the screen and count backwards from 10 to 1 as the numbers change for a total of 10 s. The baseline blocks are to increase the difficulty of the episodic memory and to simulate episodic memory in real life where intervening events occur between encoding and retrieval.

Participants then perform a retrieval block, which comprises of eight trials for a total of 80 s, with each trial lasting for 10 s. As with the encoding block, each trial of the retrieval block also begins with a 5 s presentation of an image, followed by a 5 s blank screen. The eight images scenes presented in the retrieval blocks are the same as those of the encoding blocks, but the locations of the objects in some of the scenes are altered, as illustrated in Figure 1b. Participants are asked to judge whether the location of the object in the viewed scene has changed compared with that shown in the encoding block for that scene. If the location changed, participants are required to press the right key on the MRI response box; if not, they are required to press the left key.

The retrieval block is followed by another baseline block of counting backwards before the run of another task.

The duration of the task is 550 s (including the time for the presentation of prompts), and comprises three encoding blocks, three counting backwards blocks, and three retrieval blocks. The encoding phase and retrieval phase both consist of 24 trials for analysis. Twenty‐four different scenes were used, and there were 24 different objects (people or cars). Prior to fMRI scanning, all the participants had a training session outside the MRI scanner in order to become familiar with the task. The procedure of the task is illustrated in Figure 1a,b.

#### 2.1.2 Reward‐Location Memory Task

It is clear that reward/non‐reward information reaches the hippocampus and can be part of an episodic memory (Rolls and Xiang 2005; Rolls 2022; Rolls et al. 2022c). To investigate how this affective input related to reward may be incorporated into human hippocampal function, a reward‐location memory task was used, based on the paradigm described by Rolls and Xiang (2005). Each run of the task includes an encoding/storage block, a baseline counting backwards block, and a retrieval/recall block. To begin the task, participants are instructed to fixate their gaze on a cross at the center of the screen for 10 s (Figure 1c). The encoding block consists of eight trials lasting a total of 80 s, with each trial lasting 10 s. Each trial begins with an image of a scene shown for 5 s, followed by a blank screen for 5 s as the interstimulus interval. The images are scenes of landscapes, with two locations marked by black solid circles. As illustrated in Figure 1b (left), “¥10” and “¥2” are written in white letters on the two black solid circles, respectively, representing the associations between the location and the big (¥10) reward or the small (¥2) reward. Participants are required to remember the locations of the big and small rewards in the scene of the picture, and to press a right key on the MRI response box with each stimulus presentation during a trial.

In the following baseline block, participants fix their eyes on the numbers shown in the center of the screen and count backwards from 10 to 1 as the numbers change for a total of 10 s.

In the retrieval block, with the same timing as the encoding block, the eight scenes are the same as those shown in the encoding block, but with a red circle in the scenes, as shown in Figure 1c (right). The red circle is located randomly at one of the two marked locations in the picture of the encoding block. Participants are asked to judge whether the location of the red circle represents a big or a small reward in the encoding block. If it represents a big reward, participants should press the right key on the MRI response box; if it represents a small reward, they should press the left key.

#### 2.1.3 Word Pair Association Episodic Memory Task

Another type of episodic memory is language‐related in humans, and there is evidence that the left human hippocampus is more involved with semantic information while the right human hippocampus is more involved for spatial information (Maguire and Frith 2003; Rosazza et al. 2009; Howard et al. 2011; Dalton, Hornberger, and Piguet 2016; Glikmann‐Johnston et al. 2020). To investigate the activations and pathways involved in verbal memory, we designed a word pair association memory task adapted from the verbal paired associates (VPA) task (Glikmann‐Johnston et al. (2020); Clark, Kim, and Maguire (2018)). The VPA task is widely used for verbal memory tests and has been a subtest within the Wechsler Memory Scale (WMS) from its initial version (Wechsler 1945) to the latest (WMS‐IV [Wechsler 2009]). The timing of the task is the same as the other two tasks, with three runs, and each run consisting of eight word pairs for encoding, a baseline block, and then a retrieval block. The word pairs used by Clark, Kim, and Maguire (2018) involve scene words, object words, and very low imagery abstract words which were initially sourced from databases created by Brysbaert and colleagues, which provided ratings for concreteness, word frequency, age of acquisition, valence, and arousal (Kuperman, Stadthagen‐Gonzalez, and Brysbaert 2012; Warriner, Kuperman, and Brysbaert 2013; Brysbaert, Warriner, and Kuperman 2014; van Heuven et al. 2014). The word pair associates memory task we implemented used the very low imagery abstract words used in Clark, Kim, and Maguire (2018), which were translated into Chinese double‐character abstract nouns, and then word pairs were generated randomly. The number of words with similar meanings and homophones was controlled as much as possible.

During the encoding period, participants are required to remember the eight word pairs, and to press a right key on the MRI response box with each stimulus presentation to indicate they have learned the word pair (Figure 1d).

In the retrieval block, three types of word pairs were used: the studied word pairs; recombined word pairs; and new word pairs. The recombined word pairs are random new associates constructed by words that were shown in the encoding phase, and the new word pairs had not been shown before. The recombined word pairs and new word pairs are both regarded as unstudied word pairs. Participants were asked to judge whether the word pair shown on the screen was one of the word‐pairs shown in the encoding phase. If it is a studied word pair, the participants press the right key on the MRI response box; if it is an unstudied word pair (a recombined word pair or a new word pair), the participants press the left key.

### 2.2 Neuroimaging Procedures

The fMRI neuroimaging was performed on a 3T Siemens Prisma at the Zhangjiang International Brain Imaging Centre, Fudan University, using a 32‐channel head coil. The acquisition parameters were modelled on prior investigations that aimed to minimize signal loss and distortion in the orbitofrontal cortex, anterior cingulate cortex, and amygdala (Deichmann et al. 2002, 2003; Rolls, Kellerhals, and Nichols 2015). After extensive optimization procedures, we found that imaging in approximately the plane of the Sylvian fissure met the requirements for high signal to noise ratio in these brain regions.

The neuroimaging data acquisition was optimized to adhere to the HCP's multimodal minimal preprocessing pipeline (Glasser, Smith, et al. 2016). For T1‐weighted structural imaging, we employed an MPRAGE sequence with a voxel size of 0.8‐mm isotropic, FOV of 224 mm, matrix of 320, 256 sagittal slices per slab, TR of 2400 ms, TE of 2.02 ms, TI of 1000 ms, FA of 8°, BW of 270 Hz per pixel, and ES of 7.6 ms. For T2‐weighted images, we employed a SPACE sequence with identical matrix, FOV, and slices, TR of 3200 ms, and TE of 564 ms. The parameters for all the functional scans were as follows: TR = 0.8 s, TE = 37.0 ms, and 2.0 mm isotropic resolution (using a multiband factor of 8, FA = 52°, AP phase encoding direction). Additionally, two spin echo field maps (AP and PA phase encoding directions) with the same matrix dimensions and resolution as the functional scans were acquired to aid in distortion correction of functional images.

### 2.3 Neuroimaging Data Preprocessing

We utilized the minimal preprocessing pipeline (Glasser et al. 2013) developed by the HCP to preprocess the neuroimaging data. Implemented via singularity‐based QuNex container (Version 0.95.2) (Ji et al. 2023), this preprocessing pipeline consisted of five sub‐pipelines: PreFreeSurfer, FreeSurfer, PostFreeSurfer, fMRIVolume, and fMRISurface. The PreFreeSurfer pipeline corrected and prepared the structural images (T1w and T2w), including bias field correction and MNI registration. Subsequently, the FreeSurfer pipeline segmented the volumetric images into structures required for cortical and subcortical separation. Finally, the PostFreeSurfer pipeline prepared NIFTI and GIFTI images for further analysis. For functional data analysis, fMRIVolume corrected spatial distortions and participant movement, registered functional data to structural images, and normalized to the MNI152 space. The fMRISurface step registered the surface onto the 32k Conte69 mesh, converting volumetric data into 91,282 CIFTI grayordinate space.

More details about the procedure that was used are as follows (Ji et al. 2023). FreeSurfer's recon‐all pipeline was used to segment brain‐wide gray and white matter to produce individual cortical and subcortical anatomical segmentations (Reuter et al. 2012). Cortical surface models were generated for pial and white matter boundaries as well as segmentation masks for each subcortical gray matter voxel. The T2w image was used to refine the surface tracing. Using the pial and white matter surface boundaries, a “cortical ribbon” was defined along with corresponding subcortical voxels, which were combined to generate the neural file in the Connectivity Informatics Technology Initiative (CIFTI) volume/surface “grayordinate” space for each individual participant (Glasser et al. 2013). BOLD data were motion‐corrected by aligning to the middle frame of every run via FLIRT in the initial NIFTI volume space. Next a brain‐mask was applied to exclude signal from non‐brain tissue. Next, cortical BOLD data were converted to the CIFTI gray matter matrix by sampling from the anatomically‐defined gray matter cortical ribbon and subsequently aligned to the HCP atlas using surface‐based nonlinear deformation (Glasser et al. 2013).

### 2.4 Participants

The participants were students at Fudan University, Shanghai, aged between 18 and 30 (mean 22 years). Ethical permission was obtained for the study from the Research Ethics Committee of Fudan University (ref FE231611), was performed in accordance with the Declaration of Helsinki, written information about the study was provided to participants before any scanning, and informed written consent was provided by all participants.

Neuroimaging data were available for 23 participants (14 females, 9 males) who completed the tasks with good performance following a pre‐training session of approximately 20 min that enabled good performance by all participants in the scanner (81.3% in the object‐location task, 82.1% in the reward‐location task, and 72.8% in the word pair associates task). The performance and difficulty of the first two tasks was thus closely matched, and the word‐pair task was a little more difficult, but the aim of the investigation was to investigate possible differences and similarities of the sets of 360 cortical regions activated by the different tasks, rather than to investigate general effects of task difficulty.

### 2.5 Calculation of Mean BOLD Signal Level and Functional and Effective Connectivity

The current study employed surface‐based timeseries data for the episodic memory tasks. We parcellated the timeseries data into the 360 cortical regions defined by the surface‐based HCP‐MMP atlas (Glasser, Coalson, et al. 2016; Glasser, Smith, et al. 2016). We extracted the 696 data point timeseries for each run of each task. The average timeseries for each cortical region were calculated across the three runs for each task separately for storage and recall.

The timeseries were detrended for the calculation of the activations with the Matlab function detrend, and filtered with a second order Butterworth filter set to pass frequencies close to 0.1 Hz to capture the rising and falling of the BOLD signal produced by the experimental paradigm in which a 5‐s image was followed by a 5‐s blank screen. The activations in each of the 360 cortical regions were measured by the root mean square successive difference (RMSSD) method (Nomi et al. 2017; Zhang et al. 2022), which measured the large changes in the BOLD signal produced by the stimulation paradigm of 5 s for each image followed by 5 s of a blank. The RMSSD measure produced values that correlated approximately 0.85 with the standard deviation of the BOLD signal. The activation values shown in the Figures are thus the activations averaged across all voxels within each cortical region.

Additionally, the FC matrices for each participant were constructed by assessing the Pearson correlation between the 180 cortical regions in each hemisphere and filtering of 0.008–0.08 Hz.

The effective connectivity, described in detail elsewhere (Rolls et al. 2022c, 2023a; Rolls, Deco, Zhang, et al. 2023), was calculated as described in the Supporting Information. The method involves comparing the functional connectivity between all cortical regions with the functional connectivity when there is a delay *tau* in the measurement of functional connectivity between pairs of cortical regions. When measuring effective connectivity with the fast neuroimaging modality magnetoencephalography, *tau* can be short, for example, 20 ms, and the directionality measured is as expected, for example, from the primary visual cortex V1, to V2, to V3, to V4, to ventromedial visual cortical regions VMV1–3, and to the fusiform face cortex (FFC) (Rolls, Deco, Zhang, et al. 2023; Rolls, Yan, et al. 2024). It is consistent that the propagation time from cortical region to cortical region in a hierarchy is in the order of 15–20 ms (Rolls 1992, 2023d; Panzeri et al. 2001; Rolls, Deco, Zhang, et al. 2023; Rolls, Zhang, and Feng 2024). However, when measuring effective connectivity with the much slower imaging modality fMRI, *tau* needs to be longer, typically 2000 ms as here, and then it has been found that the directionality of all effective connectivities needs to be reversed in order to match the directionality as measured with magnetoencephalography, and to be consistent with the mainly serial more strongly forward anatomical connections from, for example, V1 to V2, to V3, to V4, to ventromedial visual cortical regions VMV1–3, and to the FFC (Rolls 2023d). Possible reasons for the need to reverse the directionality for fMRI is that with *tau* needing to be as long as 2000 ms in order to measure a difference in the delayed functional connectivity, the signal propagates within 100–200 ms to higher cortical regions including those that can implement short‐term memory, and then for the remainder of the 2000 ms, the signal is propagating backwards from these higher cortical regions to earlier cortical regions using the top‐down backprojection connections (Rolls 2023d; Rolls, Deco, Zhang, et al. 2023; Rolls, Yan, et al. 2024). This correction for reversal of directionality has been incorporated in all of our fMRI studies on effective connectivity (Rolls et al. 2022a, 2022c, 2022b, 2023b, 2023a, 2023d, 2023c; Rolls, Rauschecker, et al. 2023; Rolls, Wirth, et al. 2023; Rolls, Deco, et al. 2024), including the present investigation. Interestingly, and consistently, the same correction for directionality is also needed for fMRI data from a completely different source, the anesthetized macaque (unpublished findings of E. T. Rolls using data available from Z. Wang et al. provided in the Primate Data Exchange https://fcon_1000.projects.nitrc.org/indi/indiPRIME.html). The maximum value of the effective connectivity was set to 0.2, as described elsewhere (Rolls et al. 2022c, 2023a; Rolls, Deco, Zhang, et al. 2023; Rolls, Yan, et al. 2024).

### 2.6 Statistical Analyses

Specified a priori hypotheses as follows were tested for whether activations in different potentially relevant cortical systems were different for the object‐location, reward‐location, and word‐pair episodic memory tasks during storage, and during storage compared to recall. These systems were identified in investigations always involving participants different from those in the present investigation, with many utilizing HCP data. One system relevant to the object‐location and reward‐location tasks was the ventromedial visual cortical stream implicated in spatial view functions leading through the retrosplenial scene area to the hippocampus, and consisted in the HCP‐MMP atlas of VMV1, VMV2, VMV3, VVC, PHA1, PHA2, and PHA3 (Rolls 2023d, 2023c, 2024c; Rolls et al. 2023a; Rolls, Deco, Zhang, et al. 2023; Rolls, Yan, et al. 2024). The statistical test involved a paired *t*‐test across the 23 participants of the mean activation taken across these regions of interest during the object‐location compared to the word‐pair episodic memory task. Another system, relevant to the reward‐location task, consisted of a region of interest that included reward‐related regions in the ventromedial prefrontal cortex (10d, 10pp, a10p, p10p); pregenual anterior cingulate cortex (d32, p24, s32); and medial orbitofrontal cortex (pOFC, 11l, OFC) (Rolls et al. 2022c; Rolls 2023d, 2023a), and the paired *t*‐test was between the reward‐location versus object‐location tasks. Another system, relevant to the word‐pair episodic memory task, consisted of a region of interest that included a set of temporal lobe regions involved in semantic representations (STSGa, STSda, STSdp, STSvp, TGd, TGv, and PSL [Rolls et al. 2022a]). Another system, relevant to the word‐pair episodic memory task, consisted of a region of interest that included the three closely connected regions in the HCP‐MMP atlas in Broca's area (44, 45, and 47l) (Rolls et al. 2022a). Because several prespecified tests were performed, the criterion for significance could be taken as *p* < 0.02 two‐tailed, though most effects reported were more significant than this. For the effective connectivity analyses, a data split of the *n* = 23 participants' data shown in Figures 5 and 6 was used to show that the effective connectivities across the 360 cortical regions of the two halves (*n* = 11 and *n* = 12) were correlated 0.8.

## 3 Results

### 3.1 Activations During Storage in the Object‐Location, Reward‐Location, and Word‐Pair Episodic Memory Tasks

**FIGURE 2:**
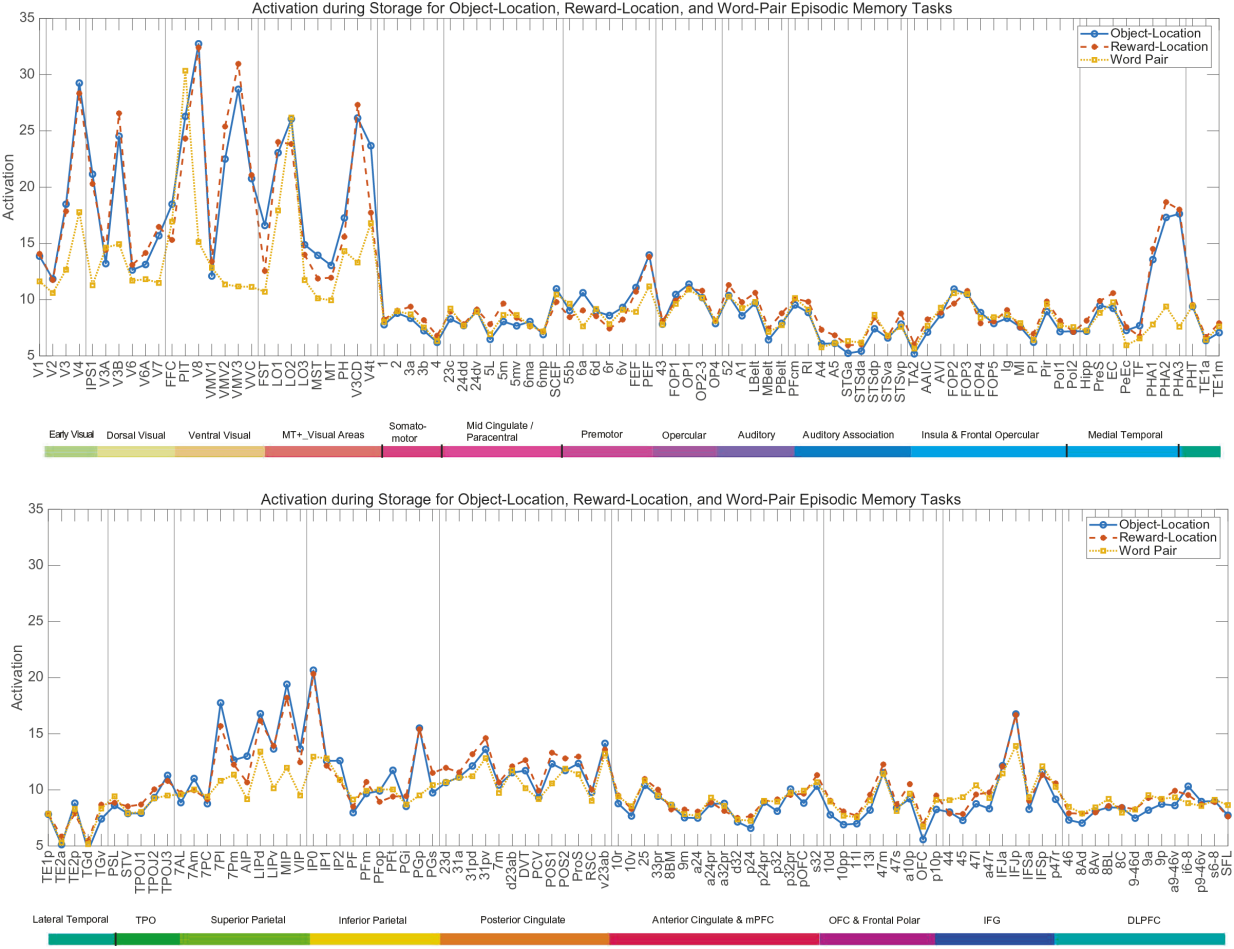
Activations in different cortical regions in the object‐location, reward‐location, and word pair episodic memory tasks during Storage. The activations are shown for the 180 regions in the surface‐based HCP‐MMP cortical parcellation (Glasser et al. 2016a), and are the mean for the left and right hemispheres. The first 90 cortical regions are in the upper panel, and the second 90 in the lower panel. The order of the cortical regions is that used in the HCPex version of the HCP‐MMP atlas (Huang et al. 2022). The abbreviations for the cortical regions are shown in Table S1, and the cortical regions are shown in Figure S1.

The activations in different cortical regions in the object‐location, reward‐location, and word‐pair episodic memory tasks during Storage are shown in Figure 2. Interesting differences in the activations in the different tasks included the following, which are in line with prior hypotheses. In the word‐pair task, the activations in a set of temporal lobe regions involved in semantic representations (STSGa, STSda, STSdp, STSvp, TGd, TGv, and PSL [Rolls et al. 2022a]) was higher than in the object‐location and reward‐location tasks (*t* = 3.6, df = 22, *p* < 0.002). In the word‐pair task, the activations in the inferior frontal cortex regions that are in Broca's area on the left (44, 45, and 47l) are higher than in the object‐location task (*t* = 4.1, df = 22, *p* < 0.0005) (with a similar result for the reward‐location vs. word‐pair episodic memory task). In contrast, the activations in the object‐location and reward‐location tasks are higher than in the word‐pair tasks in key regions in the ventromedial visual cortical pathway (VMV1, VMV2, VMV3, VVC, PHA1, PHA2, and PHA3), where the retrosplenial scene area is located (Sulpizio et al. 2020; Rolls, Yan, et al. 2024), *t* = 13.6, df = 22, *p* < 10^−8^ for the object‐location task versus the word‐pair task. Activations are also higher in these two location‐in‐scene tasks than in the word‐pair task in key intraparietal cortex regions LIPv, MIP, and VIP (*t* = 3.9, df = 22, *p* < 0.001).

Activations were higher during storage in the reward‐location than in the object‐location task in the orbitofrontal cortex regions of interest (d32, p24, pOFC, s32, 10d, 10pp, 11l, a10p, OFC, p10p, *t* = 3.3, df = 22, *p* < 0.005).

### 3.2 Activations During Recall in the Object‐Location, Reward‐Location, and Word‐Pair Episodic Memory Tasks

**FIGURE 3:**
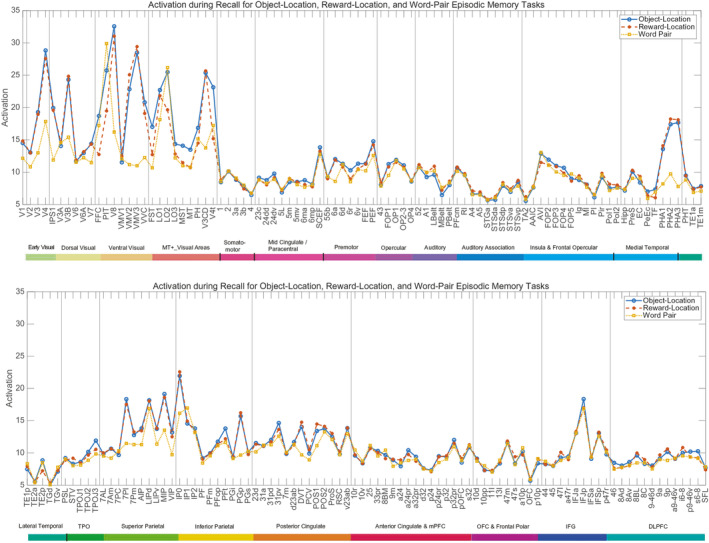
Activations for Recall for different cortical regions in the object‐location, reward‐location, and word pair episodic memory tasks. Conventions as in Figure 2.

Overall, the activations for different cortical regions during recall (Figure 3) were similar to those during storage (Figure 2), but interestingly some of the differences between the tasks were smaller during recall (Figure 3).

We start with the activations that remained task‐selective during recall (Figure 3) and almost unchanged from during storage. The activations during recall in the object‐location and reward‐location tasks are higher than in the word‐pair tasks in key regions in the ventromedial visual cortical pathway (VMV1, VMV2, VMV3, VVC, PHA1, PHA2, and PHA3), *t* = 10.6, df = 22, *p* < 10^−8^ for the object‐location task versus the word‐pair task. Activations are also higher in these two location‐in‐scene tasks than in the word‐pair task in key intraparietal cortex regions LIPv, MIP, and VIP (*t* = 4.7, df = 22, *p* = 0.0001).

For the following, the task comparisons were smaller and no longer significant in recall (Figure 3), despite the similar content of the images that were being viewed during recall to those being viewed during storage. In the word‐pair task, during recall the activations in the set of temporal lobe regions involved in semantic representations (STSGa, STSda, STSdp, STSvp, TGd, TGv, and PSL [Rolls et al. 2022a]) was no longer significantly higher than in the object‐location and reward‐location tasks (*t* = 0.5, df = 22, *p* < 0.6). Moreover, the activations were significantly higher during storage than recall (*t* = 2.2, df = 22, *p* < 0.04). In the word‐pair task, the activations in the inferior frontal cortex regions that are in Broca's area on the left (44, 45, and 47l) are not higher than in the object‐location task (*t* = 1.0, df = 22, *p* > 0.3) (with a similar result for the reward‐location vs. word‐pair episodic memory task). Moreover, the activations were significantly higher during storage than recall (*t* = 3.2, df = 22, *p* < 0.004) in regions 44, 45, and 47l. In recall, the reward‐location task activations were not higher than in the object‐location task in the orbitofrontal cortex regions of interest (d32, p24, pOFC, s32, 10d, 10pp, 11l, a10p, OFC, p10p, *t* = 0.6, df = 22, *p* > 0.5). Moreover, the activations tended to be higher during storage than recall (*t* = 2.0, df = 22, *p* < 0.057) in the orbitofrontal cortex regions of interest.

These higher activations for some task comparisons during storage versus recall of episodic memories were not due just to greater activations in the different tasks, for the correlations were low (between 0 and 0.4) between the activations and the activation differences in storage minus recall. The results were qualitatively similar for the left hemisphere alone.

Activations in some regions were higher during recall than during storage, as shown by comparing Figure 3 with Figure 2. Most prominent were intraparietal regions AIP, LIPd, IP0, IP1, and IP2, and also the SCEF, which could relate to different eye movements during recall compared to storage.

Overall, the results in this section show that the activation of some cortical systems appropriate for a particular task can be higher during the storage compared to the recall of episodic memories for the same task. This was the case for reward‐related regions in the orbitofrontal cortex, ventromedial prefrontal cortex, and pregenual anterior cingulate cortex; and for the language‐related regions in the STS and Broca's area.

### 3.3 Laterality Differences for the Activations in Different Brain Regions in the Object‐Location, Reward‐Location, and Word‐Pair Episodic Memory Tasks

**FIGURE 4:**
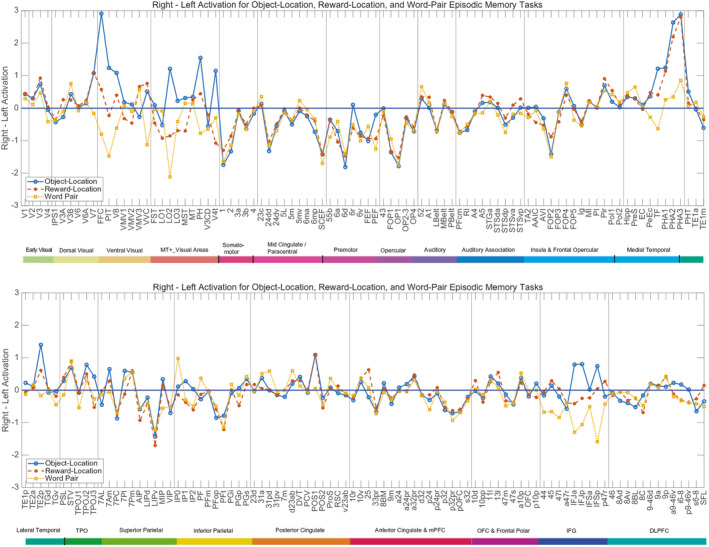
Activations for the Right Minus the Left hemisphere for different cortical regions in the object‐location, reward‐location, and word pair episodic memory tasks. The mean activation was 11.2, and activations that differ from this toward the Right are shown above that baseline, and to the Left below the baseline. Conventions as in Figure 2.

Activations for the Right minus the Left hemisphere for different cortical regions in the object‐location, reward‐location, and word pair episodic memory tasks are shown in Figure 4. The mean activation across all cortical regions was 11.2, and activations that differ from this toward the Right are shown above that baseline, and to the Left below the baseline. This shows that the laterality differences between the hemispheres can be as large as 3 parts out of 11.2 (27%), and so are relevant.

First, it is shown in Figure 4 that cortical regions involved in face and object perception, the FFC, PIT, V8, and inferior temporal TE2p, have greater activation in the right hemisphere in the object‐location memory task than in the other tasks (*p* < 0.001). In the object‐location task, faces and objects (cars) were involved in the episodic memory, and were not present in the reward‐location and word‐pair episodic memory tasks.

Second, it is shown that the right medial parahippocampal regions PHA1–3 that are implicated in spatial scene representations are more activated than in the left hemisphere during the object‐location and reward location episodic memory tasks. Interestingly, the same laterality difference was not found for the ventromedial visual cortical regions, so the lateralization for spatial scene processing in the ventromedial visual cortical stream appears to become clear at the level of the parahippocampal cortex more anterior in the temporal lobes than the ventromedial visual cortical regions (see Figures S1–S6).

Third, it is shown in Figure 4 that during the word‐pair episodic memory task, language‐related regions including Broca's area 44, 45, and 47l, and the inferior frontal gyrus regions that have high effective connectivity with them IFJa, IFJp, and IFsp (Rolls et al. 2022a) are more activated on the Left during the word‐pair association episodic memory task (*p* < 0.001). Other semantic regions (Rolls et al. 2022a) with a leftward bias include TGv, PSL, and TPOJ1.

Another interesting hemispheric difference indicated in Figure 4 is that orbitofrontal cortex region pOFC is more activated in the left hemisphere during all three memory tasks.

### 3.4 Effective Connectivities to the Hippocampal System During the Storage of Episodic Memories

Given the activations of hippocampal system and related cortical regions during the storage and recall of episodic memories shown in Figures 2, 3, 4, we next analyze the cortical pathways by which information reaches the hippocampal system during episodic memory. We focus on and start with evidence on the effective, that is directed, connectivities in the reward‐location episodic memory task, and refer to the connectivities in other tasks later.

**FIGURE 5:**
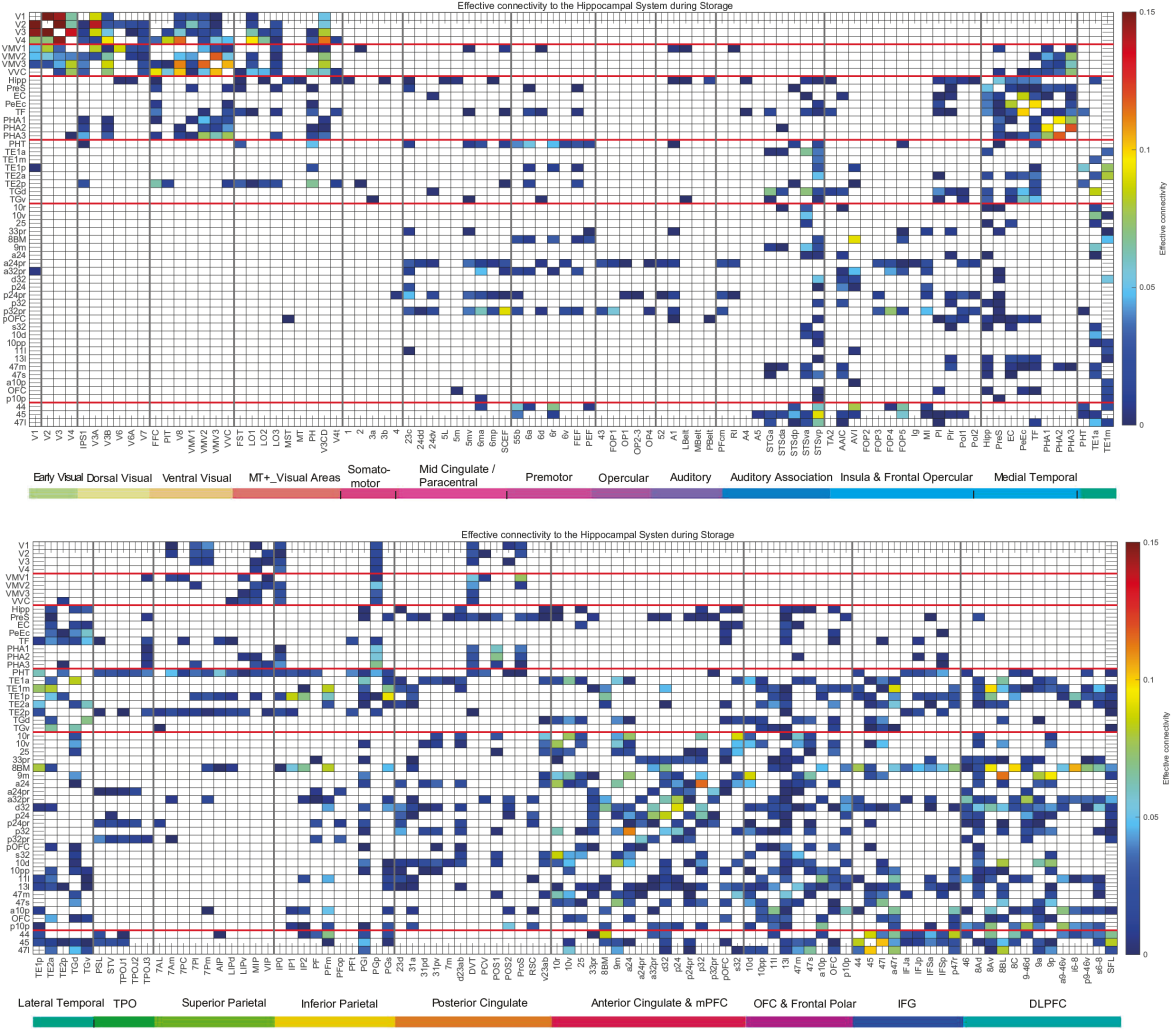
Effective connectivity to the hippocampal system and other brain regions (the rows) from all 180 regions in the HCP‐MMP parcellation (the columns) during the storage of reward‐location episodic memories. The effective connectivity strength from one cortical region to another is read from column to row. The abbreviations for the cortical regions are shown in Table S1, and the regions are shown in Figure S2. The red lines separate different groups of cortical regions, starting from the top as follows: Early visual cortical regions V1–V4; the ventromedial cortical regions VMV1–3 and VVC; the hippocampal system regions hippocampus, presubiculum, entorhinal cortex, perirhinal cortex, lateral parahippocampal region TF, medial parahippocampal regions PHA1–3; lateral temporal cortex regions and the temporal pole TGd and TGv; ventromedial prefrontal, anterior cingulate, and orbitofrontal cortex regions; and regions in Broca's area 44, 45, and 47l. The results are for the left hemisphere during the storage of reward‐location episodic memories.

Figure 5 shows the effective connectivities for the storage of reward‐location episodic memory. The connectivity from one cortical region to another is read from column to row.

**FIGURE 6:**
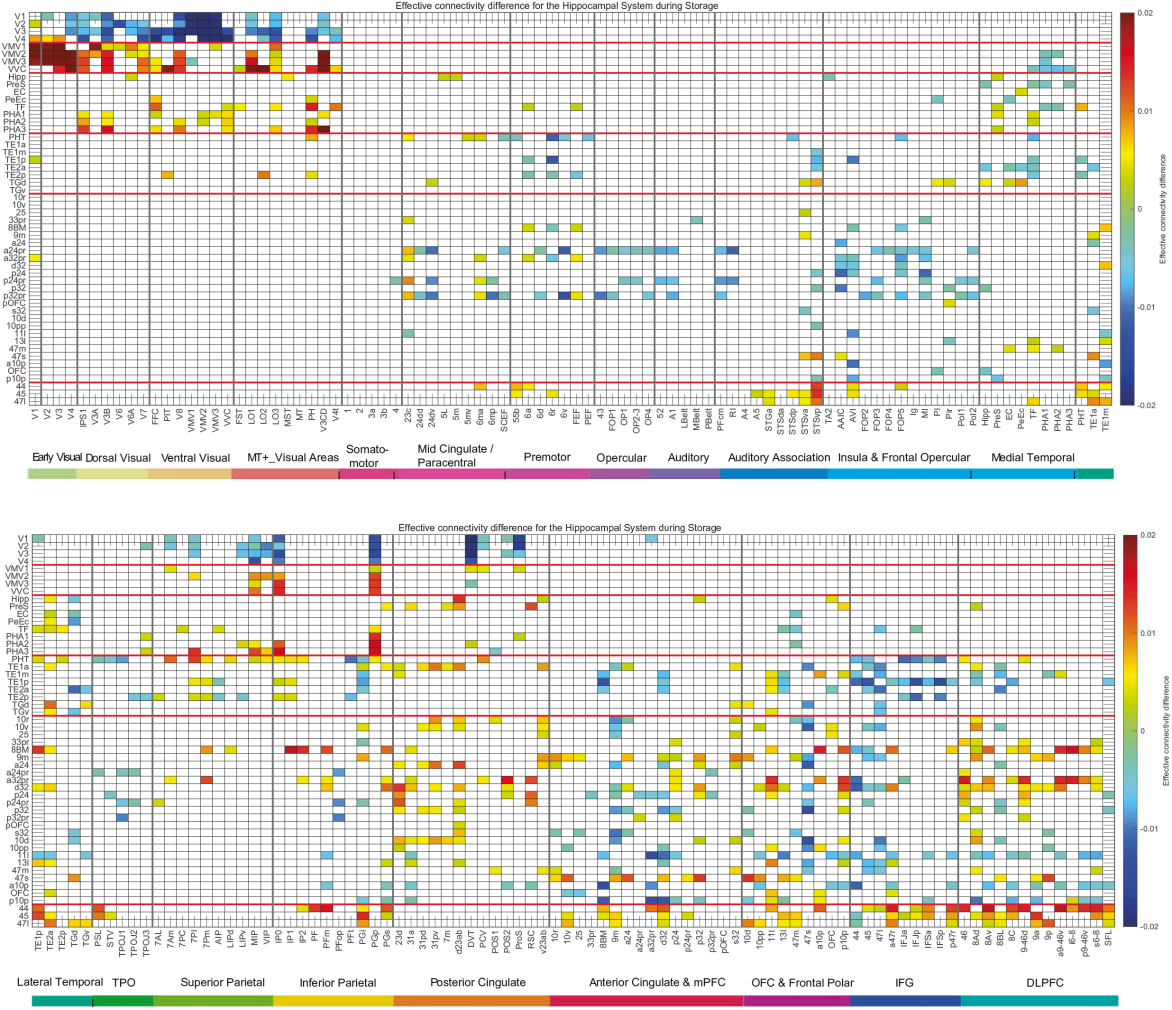
Effective connectivity directional difference between pairs of cortical regions during the storage of reward‐location episodic memories. A stronger connectivity from a column to a row is shown as positive (yellow–red), and a weaker connectivity from a column to a row is shown as negative (blue). The abbreviations for the cortical regions are shown in Table S1. The results are for the left hemisphere. Other conventions as in Figure 5.

For a spatial scene system, it is shown in Figure 5 that overall V1, V2, V4, and V4 have effective connectivity to the ventromedial visual cortical regions VMV1–3 and VVC. The connectivities in the reverse direction are weaker and can be read from row to column in Figure 5, but the differences in the two directions are brought out very clearly in Figure 6. The ventromedial visual cortical regions VMV1–3 and VVC then have connectivity to the medial parahippocampal regions PHA1–3. (The retrosplenial scene area is in the VMV and medial parahippocampal regions [Sulpizio et al. 2020; Rolls, Yan, et al. 2024] It is notable that this connectivity is stronger than from the ventromedial visual cortical regions to the lateral parahippocampal region TF, which has inputs that are stronger from the FFC in the ventrolateral visual cortical stream. The medial parahippocampal cortex PHA1–3 then has effective connectivity to the presubiculum, entorhinal cortex EC, perirhinal cortex PeEc; which in turn all have effective connectivity with the hippocampus (Figure 5).

For reward information to reach the hippocampal memory system during the reward‐location episodic memory task, it is shown in Figure 5 that the following reward/non‐reward regions have effective connectivity with the hippocampal system including the hippocampus, presubiculum, entorhinal cortex, and/or perirhinal cortex: ventromedial prefrontal 10r, subgenual cingulate 25, anterior cingulate a24, a24pr, d32, p32, p32pr, s32; and orbitofrontal cortex pOFC, 13l, 47m, and OFC.

For object information to reach the hippocampal memory system during the reward‐location episodic memory task (given that the reward stimuli were objects with numbers showing the reward value available at that location), it is shown in Figure 5 that the following object processing regions have effective connectivity with the hippocampal system including the hippocampus, presubiculum, entorhinal cortex, and/or perirhinal cortex: FFC and PIT via TE2p and then TE1p (the last mainly unimodal visual cortical object regions [Rolls, Deco, Zhang, et al. 2023]), then via the anterior temporal cortex/pole regions TE2a, TGd, and TGv, to the hippocampus, entorhinal cortex, and perirhinal cortex. This ventrolateral visual cortical object stream (Rolls et al. 2023a; Rolls 2024c; Rolls, Yan, et al. 2024) has forward effective connectivity from early cortical visual regions toward the hippocampus during the reward‐location and object‐location episodic memory tasks.

The directions in which the connectivities are greater are elucidated in Figure 6. For the spatial ventromedial visual cortical stream (Rolls 2024c), the various connectivities from V1–V4 are stronger toward the ventromedial visual cortical regions VMV1–3 and VVC. The effective connectivity algorithm is also able to show the connectivity as greater from V1 to V2; and is greater from V2 and V3 to V4 than vice versa (Figure 6), which is supportive. Further, especially VVC is shown to have stronger effective connectivity to PHA1 and PHA2; and interestingly V3CD where the occipital scene area may be located (Sulpizio et al. 2020) has stronger effective connectivity to PHA3 than vice versa. The PHA1–3 regions then have relatively equal connectivity in both directions with the presubiculum, entorhinal cortex EC, and perirhinal cortex PeEc. This is helpful new information that during the storage of reward‐spatial location episodic memories, the pathway to the hippocampus is from early visual cortical regions V1–V4 to ventromedial cortical regions VMV1–3 and VVC, and then via the medial parahippocampal gyrus PHA1–3.

For the object‐related ventrolateral visual cortical stream, Figure 6 shows that posterior inferior temporal region PIT in the FFC/V8/PIT system has forward effective connectivity to TE2p in the visual inferior temporal visual cortex, which in turn has connectivity to TF. Further, it was found that in the object‐location episodic memory task that it is FFC that has higher effective connectivity to TE2p, which has higher forward effective connectivity to the lateral parahippocampal cortex region TF, which in turn has forward effective connectivity to the hippocampus.

In relation to reward‐related processing during the reward‐location episodic memory task, it is shown in Figure 6 that medial orbitofrontal cortex reward‐related region 11l (Rolls 2023a) has stronger effective connectivity in the direction to entorhinal cortex EC; and that reward‐related pregenual anterior cingulate cortex region p32 (Rolls 2023a) has stronger effective connectivity in the direction to hippocampus and presubiculum.

In relation to communication between the hippocampus and language output systems, some effective connectivity directed away from lateral parahippocampal region TF to 47l, part of Broca's area, was evident in the reward‐location task (Figure 6), and this was more marked in the word‐pair episodic memory task from TF to both 45 and 47l.

Effective connectivity was also measured during episodic memory recall, for comparison with the effective connectivity during episodic memory storage shown in Figures 5 and 6, but significant differences between storage and recall, and the directionality of the effective connectivity, were not clearly evident. This may be because the hippocampal recall computation is relatively fast, taking perhaps 100 ms (Rolls, Zhang, and Feng 2024), so may not be easily revealed with the relatively slow neuroimaging modality of fMRI used here.

## 4 Discussion

It was found that different cortical systems to the hippocampus were activated during different types of episodic memory, and moreover that in some cases the activations were higher during the storage than the recall of episodic memory.

For object with scene location episodic memory, the activations were high in the ventromedial cortical visual stream involved in spatial processing, including the ventromedial visual cortical and medial parahippocampal system, and were similar during storage and recall. The activations for the medial parahippocampal spatial view system were higher in the right hemisphere. Activations in the face and object processing ventral visual cortical stream regions (FFC, PIT, V8, and inferior temporal TE2p) were higher in the right hemisphere during the object‐location in scene task (Figure 4).

For reward‐location in scene episodic memory, activations were also high in the ventromedial cortical visual spatial stream to the hippocampus, but were also selectively high compared to the object‐location task in storage in key reward cortical regions (ventromedial prefrontal 10r, 10v, 10d; pregenual anterior cingulate d32, p24, p32, s32; and medial orbitofrontal cortex reward‐related pOFC, 11l, OFC). These reward‐related activations were higher during storage than recall.

For word‐pair episodic memory, activations were lower in the ventromedial visual cortical/medial parahippocampal spatial stream, and were higher in language‐related regions in Broca's area (44, 45, 47l), were higher during storage than recall in these regions, and were higher in the left hemisphere for these regions and for the many highly connected inferior frontal gyrus regions in the left hemisphere.

Further, effective connectivity analyses during the episodic memory tasks showed that the direction of connectivity for these systems was from early visual cortical regions V2–V4 to the ventromedial visual cortical regions VMV1–3 and VVC for spatial scene processing; was from the pregenual anterior cingulate and orbitofrontal cortex reward systems to the hippocampal system; and was from the FFC/V8/PIT system to TE2p in the visual inferior temporal visual cortex, which has connectivity to lateral parahippocampal TF, which in turn has forward effective connectivity to the hippocampus.

### 4.1 The Ventromedial Cortical Visual Stream for Spatial Scene Processing and Episodic Memory

For the information about spatial view required for object‐scene location and reward‐scene location episodic memory, this investigation provides for the first time in such episodic memory tasks in humans in the well‐defined HCP‐MMP framework evidence that (1) the ventromedial visual cortical regions VMV2–3 and VVC and the medial parahippocampal cortex PHA1–3 are much more strongly activated in the object‐location and reward‐location episodic memory tasks than in the word‐pair memory tasks (Figure 2); (2) that for the medial parahippocampal but not ventromedial visual cortical regions the activations are stronger in the right hemisphere (Figure 4); and (3) that the direction of the effective connectivity is strongly forward from early visual cortical regions V1–V4 to the ventromedial visual cortical stream VMV1–3 and VVC regions (Figure 6). The new findings are important because they are in an episodic memory task rather than in the resting state (Rolls et al. 2023a) or when viewing stimuli in a short‐term memory 0‐back task (Rolls, Yan, et al. 2024); measure directionality that is supported by a recent investigation with the fast neuroimaging method magnetoencephalography (Rolls, Yan, et al. 2024); and are in a new group of participants independent from the HCP data that we have analyzed before (Rolls et al. 2023a; Rolls, Yan, et al. 2024). The new evidence described here also provides clear evidence that the ventromedial visual cortical and medial parahippocampal regions, which are where the retrosplenial place area (Epstein and Julian 2013; Epstein and Baker 2019) (better termed the retrosplenial scene area [Rolls 2023c; Rolls 2024c]) is located in the HCP‐MMP atlas (Sulpizio et al. 2020; Rolls, Yan, et al. 2024), are involved in the hippocampal spatial view episodic memory system; and are in a separate ventromedial cortical/medial parahippocampal visual cortical stream, in that when faces and objects are being shown, it is the ventrolateral cortical visual stream to the hippocampus via FFC, PIT, and V8 especially in the right hemisphere (Figures 2 and 4) that is also strongly activated.

This ventromedial cortical visual stream has profound implications for understanding hippocampal memory function in humans that is different from anything discovered in rodents, in that it provides evidence that “Where” representations in humans and other primates are built in a ventral visual stream where feature combinations for parts of the environment may define a location in a spatial view (Stringer, Rolls, and Trappenberg 2005; Rolls 2023d, 2023c, 2024c), completely different from “Where” place representations in rodents in which the dorsal/parietal visual stream has been invoked (Hartley et al. 2014; Bicanski and Burgess 2018). This is leading to a revolution in our understanding of the operation of the primate including human hippocampal system for episodic memory and navigation (Rolls and Treves 2024).

### 4.2 The Ventrolateral Cortical Visual Stream for Object and Face Processing and Episodic Memory

For the information about objects and faces required for object‐scene location episodic memory, this investigation provides for the first time in such episodic memory tasks in humans in the well‐defined HCP‐MMP framework evidence that visual cortical regions in the face/object ventrolateral visual cortical stream, FFC, PIT, V8, and TE2p, become selectively activated in the right hemisphere compared to other episodic memory tasks (Figure 4); and have effective connectivity that is directed from V3 and V4 to FFC, PIT, and V8, with FFC then having forward connectivity to TF and perirhinal cortex (Figure 6), which have connectivity with the hippocampus (Figure 5).

### 4.3 The Orbitofrontal and Anterior Cingulate Cortex Reward Stream to the Hippocampus and Episodic Memory

For the information about reward required for reward‐scene location episodic memory, this investigation provides for the first time in such episodic memory tasks in humans in the well‐defined HCP‐MMP framework evidence that cortical reward regions in the orbitofrontal cortex, ventromedial prefrontal cortex, and pregenual anterior cingulate cortex (Rolls 2023a) are selectively activated compared to other episodic memory tasks, and have effective connectivity that is directed toward the hippocampal episodic memory system. Although the activations were not much larger for these cortical regions in the reward‐location task than the object‐location task, it should be noted that rewards were not actually being delivered in the reward location task: the participants just had to remember the locations of rewards in scenes.

### 4.4 Semantic and Language Systems and Episodic Memory

For the information for the word‐pair episodic memory task, the hippocampus‐related pathways that are selectively activated are very different, and include temporal lobe semantic regions STSdp, temporal pole TGv, the PeriSylvian Language region PSL and TPOJ1, and Broca's area regions 44, 45, and 47l (Figure 2); with the directionality from STS and temporal lobe semantic regions to 44, 45, and 47l (Figure 6), and with a left hemisphere bias for STSdp, 44, 45, 47l and the closely connected inferior frontal gyrus regions IFJa, IFJp, and IFSp (Figure 4). The study involved 23 Chinese students of Fudan University who could speak English, and it would be of interest to extend this to more participants, especially to those whose first language is English as that might influence the lateralization.

### 4.5 Activations During the Storage versus Recall of Episodic Memory in Humans

The higher activations in some cortical regions during storage than recall need to be considered. First, in the word‐pair task, activations in the set of temporal lobe regions involved in semantic representations (STSGa, STSda, STSdp, STSvp, TGd, TGv, and PSL [Rolls et al. 2022a]) and in the inferior frontal cortex regions that are in Broca's area on the left (44, 45, and 47l) were higher during storage than recall. One possibility is that the participants spend the 5‐s period in which the image was shown and the following 5 s of blank screen rehearsing the word associations during storage, whereas during recall once a response had been selected, no further processing was useful. The same argument might apply to the activation of the reward regions including the orbitofrontal cortex during storage versus recall in the reward‐spatial location task. However, for the spatial location cortical regions in the ventromedial cortical visual stream, the activations were large and similar during storage and recall, perhaps helping to emphasize the importance of scene processing for much hippocampal episodic memory function.

The higher activations during recall than during storage found in the intraparietal regions AIP, LIPd, IP0, IP1, and IP2, and also the SCEF, could relate to different eye movements during recall compared to storage. In this investigation, visual stimuli were present during recall as well as during storage (see Figure 1). If during recall of a memory no visual stimulus is shown, the differences between recall and storage that can then be found (Favila, Lee, and Kuhl 2020) may relate to the absence during recall of all the low‐level detail of visual images that is represented in early cortical visual regions.

It may be emphasized that the aim of this investigation was to compare the cortical activations during three types of episodic memory task, as set out in the Section 1, and was not to compare the activations involved in purely perceptual processing with no episodic memory task, for which a different design would be needed. For comparison though, activations to scenes, faces, tools, and body parts using 956 HCP participants performing a simpler task that may be closer to perceptual, 0‐back working memory, are shown elsewhere (Rolls, Zhang, et al. 2024) on surface views of the HCP‐MMP atlas, and do show for example some of the scene and object regions that were activated in the present investigation. The Figures in that paper may be useful to refer to, in order to help visualize some of the cortical regions that showed activation in the present investigation. However, the visual stimuli used in the present investigation were different, as they were designed to be for object‐location or reward‐location episodic memory tasks, but our previous investigation (Rolls, Zhang, et al. 2024) does show at least the layout of some of the cortical regions in which activations are described here, and do show some of the visual stimuli that can activate them. The present study does what was intended, to compare the cortical activations during three types of episodic memory task.

### 4.6 Overall Implications

There are overall some important implications of the research described here for understanding human hippocampal function in episodic memory, including the following.

First, the hippocampus itself is equally active in the three different types of episodic memory, with no significant task‐related differences (Figure 2). This is consistent with the theory that the hippocampus provides a single network in CA3 that can enable the prototypical key components of most episodic memories to be associated together, which prototypically involve spatial with object or reward representations; temporal order; and in humans semantic/word associations (Kesner and Rolls 2015; Rolls and Treves 2024).

Second, the present research goes beyond what can be investigated in non‐human primates, by revealing the semantic pathways to and from the hippocampus that are selectively involved in word‐pair association episodic memory in humans, which very interestingly include not only anterior temporal lobe and temporo‐parieto‐occipital junction semantic regions, and Broca's area 44, 45, and 47l, but also a whole set of inferior frontal gyrus regions including IFJa, IFJp, and IFSp especially in the left hemisphere (Figure 6) which it is suggested become involved because of the major computational requirements for syntax (Rolls et al. 2022a) in which local attractor networks may play key roles (Rolls and Deco 2015; Rolls 2023d).

Third, the present research shows how each type of episodic memory task recruits different cortical regions in addition to in all cases the common denominator the hippocampus (Figures 2 and 3). In both types of spatial view episodic memory task, the ventromedial visual cortical and medial parahippocampal regions are selectively activated. In the object/face‐spatial view episodic memory task, in addition to spatial representations, object/face representations in the ventrolateral visual cortical stream via FFC to inferior temporal visual cortex TE2p are recruited. In the reward‐spatial view location task, the orbitofrontal cortex/ventromedial prefrontal cortex/pregenual anterior cingulate cortex reward system (Rolls et al. 2022c; Rolls 2023a) was recruited, especially during storage (Figure 2).

Fourth, the present research supports evidence that in humans and other primates episodic memory typically involves spatial scene information reaching the hippocampus, for the humans were always in one place, but were viewing different scenes and associating objects, faces, and rewards with different locations in viewed spatial scenes. In the rodent model, it is the place where the rodent is located that activates place cells (O'Keefe 1979; Burgess and O'Keefe 1996; O'Keefe et al. 1998; Burgess et al. 2000; Moser, Moser, and McNaughton 2017; Rolls 2023c), and that is not relevant to the prototypical types of episodic memory investigated here in which place was held constant and spatial view cells are implicated (Rolls, Robertson, and Georges‐François 1997; Robertson, Rolls, and Georges‐François 1998; Rolls et al. 1998; Georges‐François, Rolls, and Robertson 1999; Rolls and Xiang 2005, 2006; Rolls, Xiang, and Franco 2005; Rolls 2023b, 2023c; Rolls and Treves 2024).

Fifth, the present research provides important new evidence on the connectivity of the human hippocampus during episodic memory, by showing that in the ventromedial visual cortical stream during episodic memory, there is forwardly directed connectivity from early visual cortical regions V2–V4 to ventromedial visual cortical regions VMV1–3 and VVC, which in turn have effective connectivity with the medial parahippocampal cortex (Figure 6) where scene areas are located (Figure 2).

## Author Contributions

Edmund T. Rolls designed and supervised the research, performed all the analyses, led the neuroimaging data acquisition, made most of the Figures, and wrote the paper. Ruohan Zhang helped to program the task, to collect the fMRI data, and to perform the parcellation and made Figure 1. Gustavo Deco provided the code for the effective connectivity measurement. Deniz Vatansever performed the preprocessing with the HCP pipeline and set up the surface‐based parcellation of the fMRI data into surface HCP‐MMP space. Jianfeng Feng performed the funding acquisition. All authors approved the paper.

## Ethics Statement

The study received ethical approval from the Ethics Committee of the Institute of Science and Technology for Brain Inspired Intelligence at Fudan University (reference number AF/SC).

## Conflicts of Interest

The authors declare no conflicts of interest.

## Supporting information


**Data S1.** Supporting Information.

## Data Availability

Standard Matlab functions were used to calculate the functional connectivity, and to perform the paired t‐test analyzes. Basic code for the Hopf generative effective connectivity algorithm is available at https://github.com/decolab/gec. The fMRI data are available on request.
