# Supplementary material for: Selective Brain Activations and Connectivities Related to the Storage and Recall of Human Object‐Location, Reward‐Location, and Word‐Pair Episodic Memories
Source: Hum Brain Mapp. 2024 Oct 22;45(15):e70056. doi: 10.1002/hbm.70056 (PMC11494686; doi:10.1002/hbm.70056)
Supplement: Supplementary file 1 — Data S1. Supporting Information. [file HBM-45-e70056-s001.docx]

**Selective brain activations and connectivities related to the storage and recall of human object-location, reward-location, and word-pair episodic memories**

**Supplementary Material**

Edmund T Rolls^1,2,3,*^, Ruohan Zhang^1^, Gustavo Deco^4,5^, Deniz Vatansever^2^, and Jianfeng Feng^1,2^

1. Department of Computer Science, University of Warwick, Coventry, CV4 7AL, UK
2. Institute of Science and Technology for Brain Inspired Intelligence, Fudan University, Shanghai 200403, China
3. Oxford Centre for Computational Neuroscience, Oxford, UK
4. Center for Brain and Cognition, Computational Neuroscience Group, Department of Information and Communication Technologies, Universitat Pompeu Fabra, Roc Boronat 138, Barcelona, 08018, Spain; Brain and Cognition, Pompeu Fabra University, Barcelona, 08018, Spain.
5. Institució Catalana de la Recerca i Estudis Avançats (ICREA), Universitat Pompeu Fabra, Passeig Lluís Companys 23, Barcelona, 08010, Spain.

*Corresponding author:

Professor Edmund T. Rolls,

Department of Computer Science, University of Warwick, Coventry CV4 7AL, UK.

Email: [Edmund.Rolls@oxcns.org](mailto:Edmund.Rolls@oxcns.org) URL: [https://www.oxcns.org](https://www.oxcns.org/)

https://orcid.org/0000-0003-3025-1292

**Modified ordering of the HCP-MMP atlas**

The atlas used to define brain regions was the HCP-MMP surface-based atlas (Glasser, et al., 2016), illustrated in Fig. S1. In the HCP-MMP atlas, each region has its RegionID, which we show in Table S1. Detailed information about the regions is available in the Supplementary Material File NIHMS68870-supplement-Neuroanatomical_Supplementary_Results.pdf provided by Glasser et al (2016). In that Supplementary Material file, a grouping of the regions is suggested based on geographic proximity and functional similarities, and this grouping is shown in the column labelled CortexID in Table S1. That has led to a different ordering of the regions, which we show in Table S1, with the original regionIDs from the HCP atlas shown in the column headed ‘regionID’. This reordered version of the HCP-MMP atlas is described by Dr Dianne Patterson of the University of Arizona at <https://neuroimaging-core-docs.readthedocs.io/en/latest/pages/atlases.html>, where the following supporting files used to help generate Table S1 are available: HCP-MMP_UniqueRegionList.csv and Glasser_2016_Table.xlsx. We made file HCPMMP_CortexID_Ordering.xlsx from this, and this is available from the present authors. The connectivity matrices shown in the present paper used the ordering shown in Table S1, which is also used in the volumetric and extended form of this atlas (Huang, et al., 2022).

**Table S1.** Regions defined in the modified Human Connectome Project atlas (Glasser, et al., 2016). L=left hemisphere, R=right. The column ‘Reordered region ID’ is that used in Figs. 1-5, and is a reordering of that based on suggestions in the Supplementary Information of Glasser et al (2016). In that Supplementary Information of that paper, the 360 regions are grouped based on geographic proximity and functional similarities, which was reorganized and provided by Dr Dianne Patterson of the University of Arizona at [https://neuroimaging-core-docs.readthedocs.io/en/latest/pages/atlases.html](https://neuroimaging-core-docs.readthedocs.io/en/latest/pages/atlases.html%20) with the HCP-MMP_UniqueRegionList.csv and is shown in the column labelled CortexID in Table S1. The volumes are in mm^3^. This modified atlas with the reordering is described elsewhere (Huang, et al., 2022).

| **Reordered**  **ID (L, R)** | **Region** | **RegionLongName** | **Cortical Division** | **Cortex**  **ID** | **Original**  **ID** | **Voxel numbers (1mm^3^) (L,R)** |
| --- | --- | --- | --- | --- | --- | --- |
| 1, 181 | V1 | Primary_Visual_Cortex | Primary_Visual | 1 | 1 | 13812, 13406 |
| 2, 182 | V2 | Second_Visual_Area | Early_Visual | 2 | 4 | 9515, 9420 |
| 3, 183 | V3 | Third_Visual_Area | Early_Visual | 2 | 5 | 7106, 7481 |
| 4, 184 | V4 | Fourth_Visual_Area | Early_Visual | 2 | 6 | 4782, 4537 |
| 5, 185 | IPS1 | IntraParietal_Sulcus_Area_1 | Dorsal_Stream_Visual | 3 | 17 | 1751, 1750 |
| 6, 186 | V3A | Area_V3A | Dorsal_Stream_Visual | 3 | 13 | 2191, 2212 |
| 7, 187 | V3B | Area_V3B | Dorsal_Stream_Visual | 3 | 19 | 639, 731 |
| 8, 188 | V6 | Sixth_Visual_Area | Dorsal_Stream_Visual | 3 | 3 | 1402, 1559 |
| 9, 189 | V6A | Area_V6A | Dorsal_Stream_Visual | 3 | 152 | 904, 734 |
| 10, 190 | V7 | Seventh_Visual_Area | Dorsal_Stream_Visual | 3 | 16 | 1005, 1041 |
| 11, 191 | FFC | Fusiform_Face_Complex | Ventral_Stream_Visual | 4 | 18 | 3848, 4402 |
| 12, 192 | PIT | Posterior_InferoTemporal_complex | Ventral_Stream_Visual | 4 | 22 | 1392, 1386 |
| 13, 193 | V8 | Eighth_Visual_Area | Ventral_Stream_Visual | 4 | 7 | 1361, 1175 |
| 14, 194 | VMV1 | VentroMedial_Visual_Area_1 | Ventral_Stream_Visual | 4 | 153 | 939, 1219 |
| 15, 195 | VMV2 | VentroMedial_Visual_Area_2 | Ventral_Stream_Visual | 4 | 160 | 639, 923 |
| 16, 196 | VMV3 | VentroMedial_Visual_Area_3 | Ventral_Stream_Visual | 4 | 154 | 941, 1242 |
| 17, 197 | VVC | Ventral_Visual_Complex | Ventral_Stream_Visual | 4 | 163 | 2487, 2753 |
| 18, 198 | FST | Area_FST | MT+_Complex | 5 | 157 | 1324, 1683 |
| 19, 199 | LO1 | Area_Lateral_Occipital_1 | MT+_Complex | 5 | 20 | 619, 909 |
| 20, 200 | LO2 | Area_Lateral_Occipital_2 | MT+_Complex | 5 | 21 | 1179, 1062 |
| 21, 201 | LO3 | Area_Lateral_Occipital_3 | MT+_Complex | 5 | 159 | 438, 915 |
| 22, 202 | MST | Medial_Superior_Temporal_Area | MT+_Complex | 5 | 2 | 794, 1036 |
| 23, 203 | MT | Middle_Temporal_Area | MT+_Complex | 5 | 23 | 620, 1005 |
| 24, 204 | PH | Area_PH | MT+_Complex | 5 | 138 | 3453, 3205 |
| 25, 205 | V3CD | Area_V3CD | MT+_Complex | 5 | 158 | 876, 1222 |
| 26, 206 | V4t | Area_V4t | MT+_Complex | 5 | 156 | 1037, 1249 |
| 27, 207 | 1 | Area_1 | SomaSens_Motor | 6 | 51 | 6590, 5925 |
| 28, 208 | 2 | Area_2 | SomaSens_Motor | 6 | 52 | 4278, 4727 |
| 29, 209 | 3a | Area_3a | SomaSens_Motor | 6 | 53 | 2247, 2286 |
| 30, 210 | 3b | Primary_Sensory_Cortex | SomaSens_Motor | 6 | 9 | 5451, 4350 |
| 31, 211 | 4 | Primary_Motor_Cortex | SomaSens_Motor | 6 | 8 | 10776, 10254 |
| 32, 212 | 23c | Area_23c | ParaCentral_MidCing | 7 | 38 | 2259, 2498 |
| 33, 213 | 24dd | Dorsal_Area_24d | ParaCentral_MidCing | 7 | 40 | 2665, 2820 |
| 34, 214 | 24dv | Ventral_Area_24d | ParaCentral_MidCing | 7 | 41 | 1076, 1349 |
| 35, 215 | 5L | Area_5L | ParaCentral_MidCing | 7 | 39 | 2249, 2327 |
| 36, 216 | 5m | Area_5m | ParaCentral_MidCing | 7 | 36 | 1483, 2079 |
| 37, 217 | 5mv | Area_5m_ventral | ParaCentral_MidCing | 7 | 37 | 1651, 1996 |
| 38, 218 | 6ma | Area_6m_anterior | ParaCentral_MidCing | 7 | 44 | 3941, 4251 |
| 39, 219 | 6mp | Area_6mp | ParaCentral_MidCing | 7 | 55 | 3701, 3105 |
| 40, 220 | SCEF | Supplementary_and_Cingulate_Eye_Field | ParaCentral_MidCing | 7 | 43 | 3500, 3371 |
| 41, 221 | 55b | Area_55b | Premotor | 8 | 12 | 2422, 1537 |
| 42, 222 | 6a | Area_6_anterior | Premotor | 8 | 96 | 4233, 3752 |
| 43, 223 | 6d | Dorsal_area_6 | Premotor | 8 | 54 | 2916, 2909 |
| 44, 224 | 6r | Rostral_Area_6 | Premotor | 8 | 78 | 3029, 3981 |
| 45, 225 | 6v | Ventral_Area_6 | Premotor | 8 | 56 | 2075, 2516 |
| 46, 226 | FEF | Frontal_Eye_Fields | Premotor | 8 | 10 | 1787, 1889 |
| 47, 227 | PEF | Premotor_Eye_Field | Premotor | 8 | 11 | 1006, 1258 |
| 48, 228 | 43 | Area_43 | Posterior_Opercular | 9 | 99 | 1889, 1678 |
| 49, 229 | FOP1 | Frontal_Opercular_Area_1 | Posterior_Opercular | 9 | 113 | 879, 932 |
| 50, 230 | OP1 | Area_OP1-SII | Posterior_Opercular | 9 | 101 | 1275, 1072 |
| 51, 231 | OP2-3 | Area_OP2-3-VS | Posterior_Opercular | 9 | 102 | 943, 792 |
| 52, 232 | OP4 | Area_OP4-PV | Posterior_Opercular | 9 | 100 | 2332, 2409 |
| 53, 233 | 52 | Area_52 | Early_Auditory | 10 | 103 | 725, 580 |
| 54, 234 | A1 | Primary_Auditory_Cortex | Early_Auditory | 10 | 24 | 1023, 796 |
| 55, 235 | LBelt | Lateral_Belt_Complex | Early_Auditory | 10 | 174 | 820, 901 |
| 56, 236 | MBelt | Medial_Belt_Complex | Early_Auditory | 10 | 173 | 1242, 1236 |
| 57, 237 | PBelt | ParaBelt_Complex | Early_Auditory | 10 | 124 | 1719, 1439 |
| 58, 238 | PFcm | Area_PFcm | Early_Auditory | 10 | 105 | 1486, 1485 |
| 59, 239 | RI | RetroInsular_Cortex | Early_Auditory | 10 | 104 | 1149, 1334 |
| 60, 240 | A4 | Auditory_4_Complex | Auditory_Association | 11 | 175 | 3514, 3610 |
| 61, 241 | A5 | Auditory_5_Complex | Auditory_Association | 11 | 125 | 3346, 3881 |
| 62, 242 | STGa | Area_STGa | Auditory_Association | 11 | 123 | 2509, 2187 |
| 63, 243 | STSda | Area_STSd_anterior | Auditory_Association | 11 | 128 | 1944, 2389 |
| 64, 244 | STSdp | Area_STSd_posterior | Auditory_Association | 11 | 129 | 1994, 2605 |
| 65, 245 | STSva | Area_STSv_anterior | Auditory_Association | 11 | 176 | 1694, 1900 |
| 66, 246 | STSvp | Area_STSv_posterior | Auditory_Association | 11 | 130 | 2898, 2515 |
| 67, 247 | TA2 | Area_TA2 | Auditory_Association | 11 | 107 | 1518, 1726 |
| 68, 248 | AAIC | Anterior_Agranular_Insula_Complex | Insula_FrontalOperc | 12 | 112 | 1859, 1691 |
| 69, 249 | AVI | Anterior_Ventral_Insular_Area | Insula_FrontalOperc | 12 | 111 | 1446, 1792 |
| 70, 250 | FOP2 | Frontal_Opercular_Area_2 | Insula_FrontalOperc | 12 | 115 | 750, 720 |
| 71, 251 | FOP3 | Frontal_Opercular_Area_3 | Insula_FrontalOperc | 12 | 114 | 754, 614 |
| 72, 252 | FOP4 | Frontal_Opercular_Area_4 | Insula_FrontalOperc | 12 | 108 | 2522, 1678 |
| 73, 253 | FOP5 | Area_Frontal_Opercular_5 | Insula_FrontalOperc | 12 | 169 | 1297, 1365 |
| 74, 254 | Ig | Insular_Granular_Complex | Insula_FrontalOperc | 12 | 168 | 841, 1077 |
| 75, 255 | MI | Middle_Insular_Area | Insula_FrontalOperc | 12 | 109 | 2102, 1960 |
| 76, 256 | PI | Para-Insular_Area | Insula_FrontalOperc | 12 | 178 | 1033, 1058 |
| 77, 257 | Pir | Piriform_Cortex | Insula_FrontalOperc | 12 | 110 | 2287, 1856 |
| 78, 258 | PoI1 | Area_Posterior_Insular_1 | Insula_FrontalOperc | 12 | 167 | 1811, 1835 |
| 79, 259 | PoI2 | Posterior_Insular_Area_2 | Insula_FrontalOperc | 12 | 106 | 2747, 2675 |
| 80, 260 | H | Hippocampus | Medial_Temporal | 13 | 120 | 4283, 3626 |
| 81, 261 | PreS | PreSubiculum | Medial_Temporal | 13 | 119 | 1817, 1558 |
| 82, 262 | EC | Entorhinal_Cortex | Medial_Temporal | 13 | 118 | 2127, 2110 |
| 83, 263 | PeEc | Perirhinal_Ectorhinal_Cortex | Medial_Temporal | 13 | 122 | 4826, 4755 |
| 84, 264 | TF | Area_TF | Medial_Temporal | 13 | 135 | 3986, 4752 |
| 85, 265 | PHA1 | ParaHippocampal_Area_1 | Medial_Temporal | 13 | 126 | 1281, 1168 |
| 86, 266 | PHA2 | ParaHippocampal_Area_2 | Medial_Temporal | 13 | 155 | 783, 771 |
| 87, 267 | PHA3 | ParaHippocampal_Area_3 | Medial_Temporal | 13 | 127 | 2023, 1122 |
| 88, 268 | PHT | Area_PHT | Lateral_Temporal | 14 | 137 | 4182, 3410 |
| 89, 269 | TE1a | Area_TE1_anterior | Lateral_Temporal | 14 | 132 | 5227, 4180 |
| 90, 270 | TE1m | Area_TE1_Middle | Lateral_Temporal | 14 | 177 | 3339, 3429 |
| 91, 271 | TE1p | Area_TE1_posterior | Lateral_Temporal | 14 | 133 | 7116, 6010 |
| 92, 272 | TE2a | Area_TE2_anterior | Lateral_Temporal | 14 | 134 | 5691, 5753 |
| 93, 273 | TE2p | Area_TE2_posterior | Lateral_Temporal | 14 | 136 | 4115, 3040 |
| 94, 274 | TGd | Area_TG_dorsal | Lateral_Temporal | 14 | 131 | 10192, 10269 |
| 95, 275 | TGv | Area_TG_Ventral | Lateral_Temporal | 14 | 172 | 3694, 4515 |
| 96, 276 | PSL | PeriSylvian_Language_Area | TPO | 15 | 25 | 2154, 2759 |
| 97, 277 | STV | Superior_Temporal_Visual_Area | TPO | 15 | 28 | 2322, 2294 |
| 98, 278 | TPOJ1 | Area_TemporoParietoOccipital_Junction_1 | TPO | 15 | 139 | 2102, 3938 |
| 99, 279 | TPOJ2 | Area_TemporoParietoOccipital_Junction_2 | TPO | 15 | 140 | 1930, 2068 |
| 100, 280 | TPOJ3 | Area_TemporoParietoOccipital_Junction_3 | TPO | 15 | 141 | 1290, 1277 |
| 101, 281 | 7AL | Lateral_Area_7A | Superior_Parietal | 16 | 42 | 2134, 2030 |
| 102, 282 | 7Am | Medial_Area_7A | Superior_Parietal | 16 | 45 | 2995, 2379 |
| 103, 283 | 7PC | Area_7PC | Superior_Parietal | 16 | 47 | 3151, 3415 |
| 104, 284 | 7PL | Lateral_Area_7P | Superior_Parietal | 16 | 46 | 1695, 1363 |
| 105, 285 | 7Pm | Medial_Area_7P | Superior_Parietal | 16 | 29 | 1601, 1308 |
| 106, 286 | AIP | Anterior_IntraParietal_Area | Superior_Parietal | 16 | 117 | 1999, 2542 |
| 107, 287 | LIPd | Area_Lateral_IntraParietal_dorsal | Superior_Parietal | 16 | 95 | 1008, 869 |
| 108, 288 | LIPv | Area_Lateral_IntraParietal_ventral | Superior_Parietal | 16 | 48 | 1681, 1783 |
| 109, 289 | MIP | Medial_IntraParietal_Area | Superior_Parietal | 16 | 50 | 1872, 2403 |
| 110, 290 | VIP | Ventral_IntraParietal_Complex | Superior_Parietal | 16 | 49 | 1890, 1577 |
| 111, 291 | IP0 | Area_IntraParietal_0 | Inferior_Parietal | 17 | 146 | 1203, 1239 |
| 112, 292 | IP1 | Area_IntraParietal_1 | Inferior_Parietal | 17 | 145 | 1692, 1632 |
| 113, 293 | IP2 | Area_IntraParietal_2 | Inferior_Parietal | 17 | 144 | 2102, 1861 |
| 114, 294 | PF | Area_PF_Complex | Inferior_Parietal | 17 | 148 | 5457, 5251 |
| 115, 295 | PFm | Area_PFm_Complex | Inferior_Parietal | 17 | 149 | 8220, 8141 |
| 116, 296 | PFop | Area_PF_Opercular | Inferior_Parietal | 17 | 147 | 1797, 1783 |
| 117, 297 | PFt | Area_PFt | Inferior_Parietal | 17 | 116 | 1983, 2039 |
| 118, 298 | PGi | Area_PGi | Inferior_Parietal | 17 | 150 | 4791, 4970 |
| 119, 299 | PGp | Area_PGp | Inferior_Parietal | 17 | 143 | 2501, 3740 |
| 120, 300 | PGs | Area_PGs | Inferior_Parietal | 17 | 151 | 4552, 3366 |
| 121, 301 | 23d | Area_23d | Posterior_Cingulate | 18 | 32 | 1261, 1513 |
| 122, 302 | 31a | Area_31a | Posterior_Cingulate | 18 | 162 | 1260, 1116 |
| 123, 303 | 31pd | Area_31pd | Posterior_Cingulate | 18 | 161 | 1428, 864 |
| 124, 304 | 31pv | Area_31p_ventral | Posterior_Cingulate | 18 | 35 | 950, 1022 |
| 125, 305 | 7m | Area_7m | Posterior_Cingulate | 18 | 30 | 2128, 2067 |
| 126, 306 | d23ab | Area_dorsal_23_a+b | Posterior_Cingulate | 18 | 34 | 1607, 1106 |
| 127, 307 | DVT | Dorsal_Transitional_Visual_Area | Posterior_Cingulate | 18 | 142 | 1806, 2176 |
| 128, 308 | PCV | PreCuneus_Visual_Area | Posterior_Cingulate | 18 | 27 | 2245, 2416 |
| 129, 309 | POS1 | Parieto-Occipital_Sulcus_Area_1 | Posterior_Cingulate | 18 | 31 | 2531, 2727 |
| 130, 310 | POS2 | Parieto-Occipital_Sulcus_Area_2 | Posterior_Cingulate | 18 | 15 | 3261, 3093 |
| 131, 311 | ProS | ProStriate_Area | Posterior_Cingulate | 18 | 121 | 1222, 1055 |
| 132, 312 | RSC | RetroSplenial_Complex | Posterior_Cingulate | 18 | 14 | 2830, 3067 |
| 133, 313 | v23ab | Area_ventral_23_a+b | Posterior_Cingulate | 18 | 33 | 916, 1089 |
| 134, 314 | 10r | Area_10r | AntCing_MedPFC | 19 | 65 | 1589, 1053 |
| 135, 315 | 10v | Area_10v | AntCing_MedPFC | 19 | 88 | 3906, 2667 |
| 136, 316 | 25 | Area_25 | AntCing_MedPFC | 19 | 164 | 1911, 2135 |
| 137, 317 | 33pr | Area_33_prime | AntCing_MedPFC | 19 | 58 | 1354, 1316 |
| 138, 318 | 8BM | Area_8BM | AntCing_MedPFC | 19 | 63 | 3122, 3436 |
| 139, 319 | 9m | Area_9_Middle | AntCing_MedPFC | 19 | 69 | 6338, 5881 |
| 140, 320 | a24 | Area_a24 | AntCing_MedPFC | 19 | 61 | 2085, 2152 |
| 141, 321 | a24pr | Anterior_24_prime | AntCing_MedPFC | 19 | 59 | 1095, 1474 |
| 142, 322 | a32pr | Area_anterior_32_prime | AntCing_MedPFC | 19 | 179 | 1759, 1118 |
| 143, 323 | d32 | Area_dorsal_32 | AntCing_MedPFC | 19 | 62 | 2228, 2374 |
| 144, 324 | p24 | Area_posterior_24 | AntCing_MedPFC | 19 | 180 | 2394, 2442 |
| 145, 325 | p24pr | Area_Posterior_24_prime | AntCing_MedPFC | 19 | 57 | 1422, 1724 |
| 146, 326 | p32 | Area_p32 | AntCing_MedPFC | 19 | 64 | 1180, 1765 |
| 147, 327 | p32pr | Area_p32_prime | AntCing_MedPFC | 19 | 60 | 1569, 1305 |
| 148, 328 | pOFC | Posterior_OFC_Complex | AntCing_MedPFC | 19 | 166 | 2486, 2836 |
| 149, 329 | s32 | Area_s32 | AntCing_MedPFC | 19 | 165 | 604, 1015 |
| 150, 330 | 10d | Area_10d | OrbPolaFrontal | 20 | 72 | 3644, 3096 |
| 151, 331 | 10pp | Polar_10p | OrbPolaFrontal | 20 | 90 | 1997, 2487 |
| 152, 332 | 11l | Area_11l | OrbPolaFrontal | 20 | 91 | 3531, 3793 |
| 153, 333 | 13l | Area_13l | OrbPolaFrontal | 20 | 92 | 2429, 1757 |
| 154, 334 | 47m | Area_47m | OrbPolaFrontal | 20 | 66 | 799, 781 |
| 155, 335 | 47s | Area_47s | OrbPolaFrontal | 20 | 94 | 2795, 3080 |
| 156, 336 | a10p | Area_anterior_10p | OrbPolaFrontal | 20 | 89 | 1964, 1748 |
| 157, 337 | OFC | Orbital_Frontal_Complex | OrbPolaFrontal | 20 | 93 | 4560, 5232 |
| 158, 338 | p10p | Area_posterior_10p | OrbPolaFrontal | 20 | 170 | 2116, 2365 |
| 159, 339 | 44 | Area_44 | Inferior_Frontal | 21 | 74 | 2435, 2589 |
| 160, 340 | 45 | Area_45 | Inferior_Frontal | 21 | 75 | 3762, 2962 |
| 161, 341 | 47l | Area_47l_(47_lateral) | Inferior_Frontal | 21 | 76 | 2527, 2592 |
| 162, 342 | a47r | Area_anterior_47r | Inferior_Frontal | 21 | 77 | 4167, 3763 |
| 163, 343 | IFJa | Area_IFJa | Inferior_Frontal | 21 | 79 | 1513, 1405 |
| 164, 344 | IFJp | Area_IFJp | Inferior_Frontal | 21 | 80 | 960, 740 |
| 165, 345 | IFSa | Area_IFSa | Inferior_Frontal | 21 | 82 | 2057, 2641 |
| 166, 346 | IFSp | Area_IFSp | Inferior_Frontal | 21 | 81 | 1589, 1730 |
| 167, 347 | p47r | Area_posterior_47r | Inferior_Frontal | 21 | 171 | 2133, 1761 |
| 168, 348 | 46 | Area_46 | Dorsolateral_Prefrontal | 22 | 84 | 4863, 4394 |
| 169, 349 | 8Ad | Area_8Ad | Dorsolateral_Prefrontal | 22 | 68 | 3386, 3492 |
| 170, 350 | 8Av | Area_8Av | Dorsolateral_Prefrontal | 22 | 67 | 4807, 5902 |
| 171, 351 | 8BL | Area_8B_Lateral | Dorsolateral_Prefrontal | 22 | 70 | 3377, 4078 |
| 172, 352 | 8C | Area_8C | Dorsolateral_Prefrontal | 22 | 73 | 4085, 3134 |
| 173, 353 | 9-46d | Area_9-46d | Dorsolateral_Prefrontal | 22 | 86 | 4534, 4666 |
| 174, 354 | 9a | Area_9_anterior | Dorsolateral_Prefrontal | 22 | 87 | 3706, 3048 |
| 175, 355 | 9p | Area_9_Posterior | Dorsolateral_Prefrontal | 22 | 71 | 3426, 2488 |
| 176, 356 | a9-46v | Area_anterior_9-46v | Dorsolateral_Prefrontal | 22 | 85 | 3314, 2628 |
| 177, 357 | i6-8 | Inferior_6-8_Transitional_Area | Dorsolateral_Prefrontal | 22 | 97 | 1764, 2418 |
| 178, 358 | p9-46v | Area_posterior_9-46v | Dorsolateral_Prefrontal | 22 | 83 | 2871, 4635 |
| 179, 359 | s6-8 | Superior_6-8_Transitional_Area | Dorsolateral_Prefrontal | 22 | 98 | 1336, 2132 |
| 180, 360 | SFL | Superior_Frontal_Language_Area | Dorsolateral_Prefrontal | 22 | 26 | 3873, 3055 |

Column 1 (Reordered ID) shows the order in HCPex based on the HCP-MMP1_UniqueRegionList.csv, as described in the Methods, of the 360 cortical regions originally defined by Glasser et al (2016). The names of the cortical divisions shown in column 4 come from the same .csv file. The sixth column shows the original order used by Glasser et al (2016). Abbreviations: L=left hemisphere, R=right. MT+_Complex, MT+_Complex_and_Neighboring_Visual_Areas; SomaSens_Motor, Somatosensory_and_Motor; ParaCentral_MidCing, Paracentral_Lobular_and_Mid_Cingulate; Insula_FrontalOperc, Insular_and_Frontal_Opercular; TPO, Temporo-Parieto-Occipital_Junction; AntCing_MedPFC, Anterior_Cingulate_and_Medial_Prefrontal; OrbPolaFrontal, Orbital_and_Polar_Frontal.

Fig. S1-1. Example coronal slices showing regions defined in the HCPex atlas and added subcortical regions (Huang, et al., 2022). The abbreviations are as in Table S1. The y values for the coronal slices are in MNI coordinates.


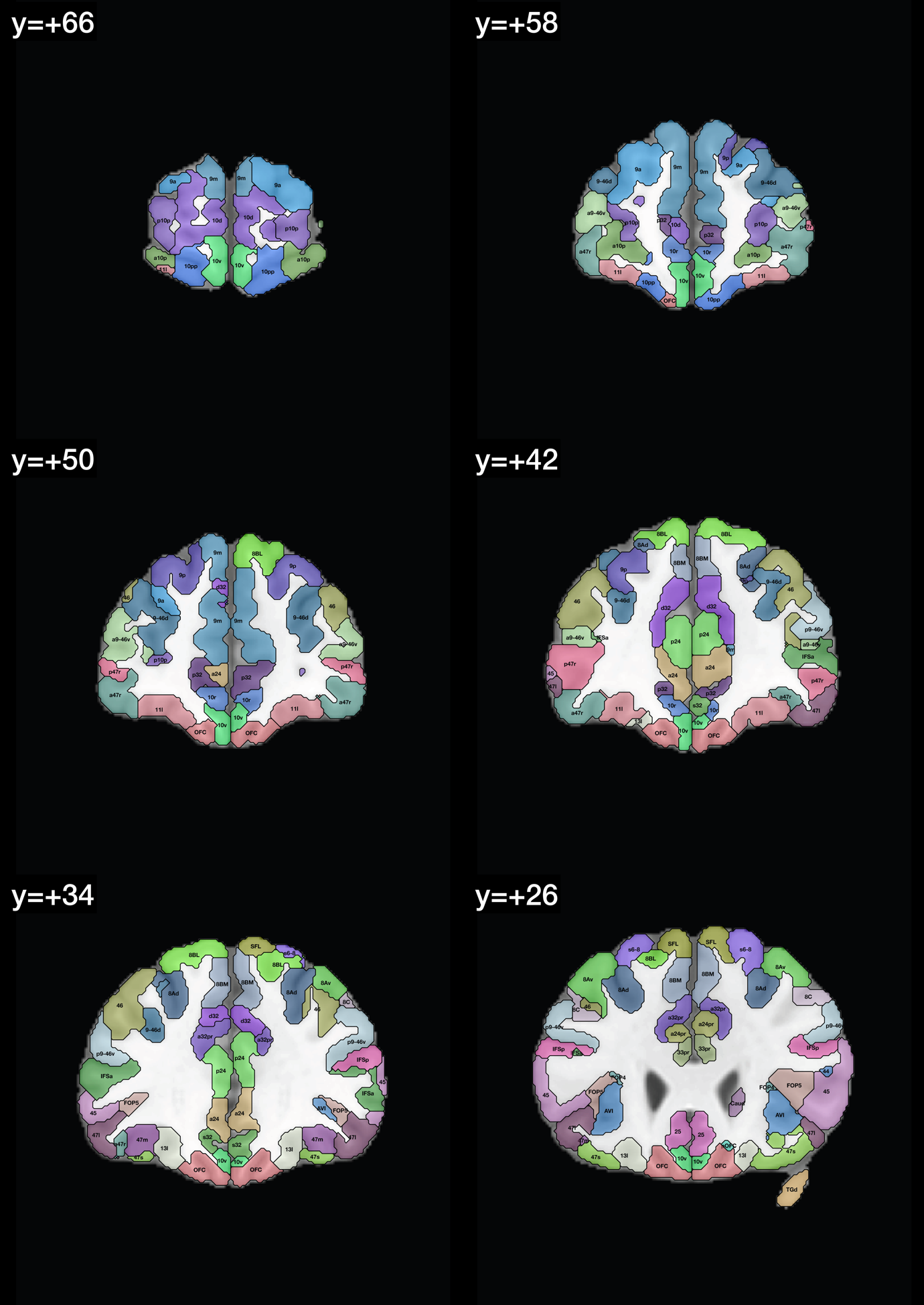


Fig. S1-2. Example coronal slices showing regions defined in the HCPex atlas and added subcortical regions (Huang, et al., 2022). The abbreviations are as in Table S1. The y values for the coronal slices are in MNI coordinates.


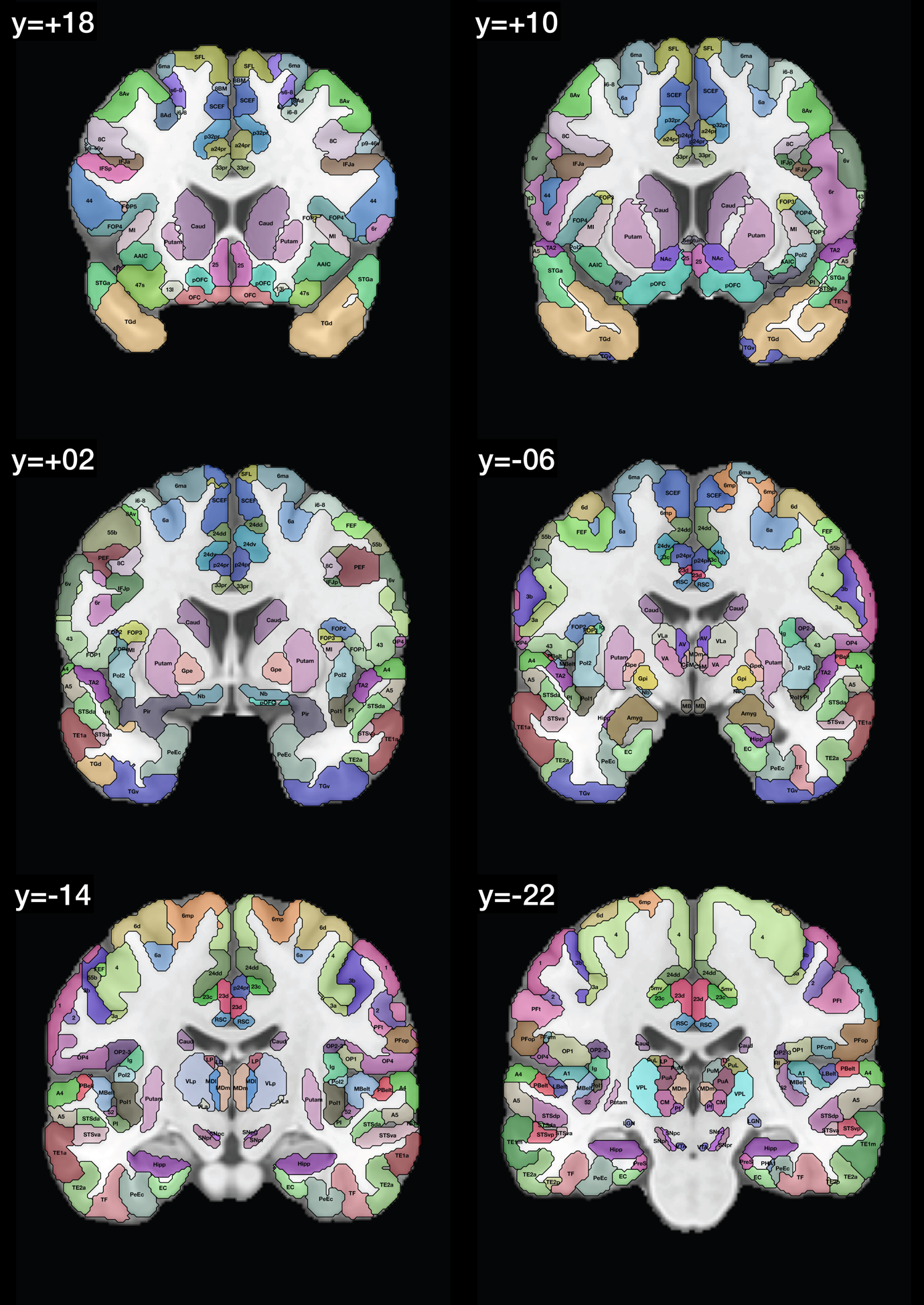


Fig. S1-3. Example coronal slices showing regions defined in the HCPex atlas and added subcortical regions (Huang, et al., 2022). The abbreviations are as in Table S1. The y values for the coronal slices are in MNI coordinates.


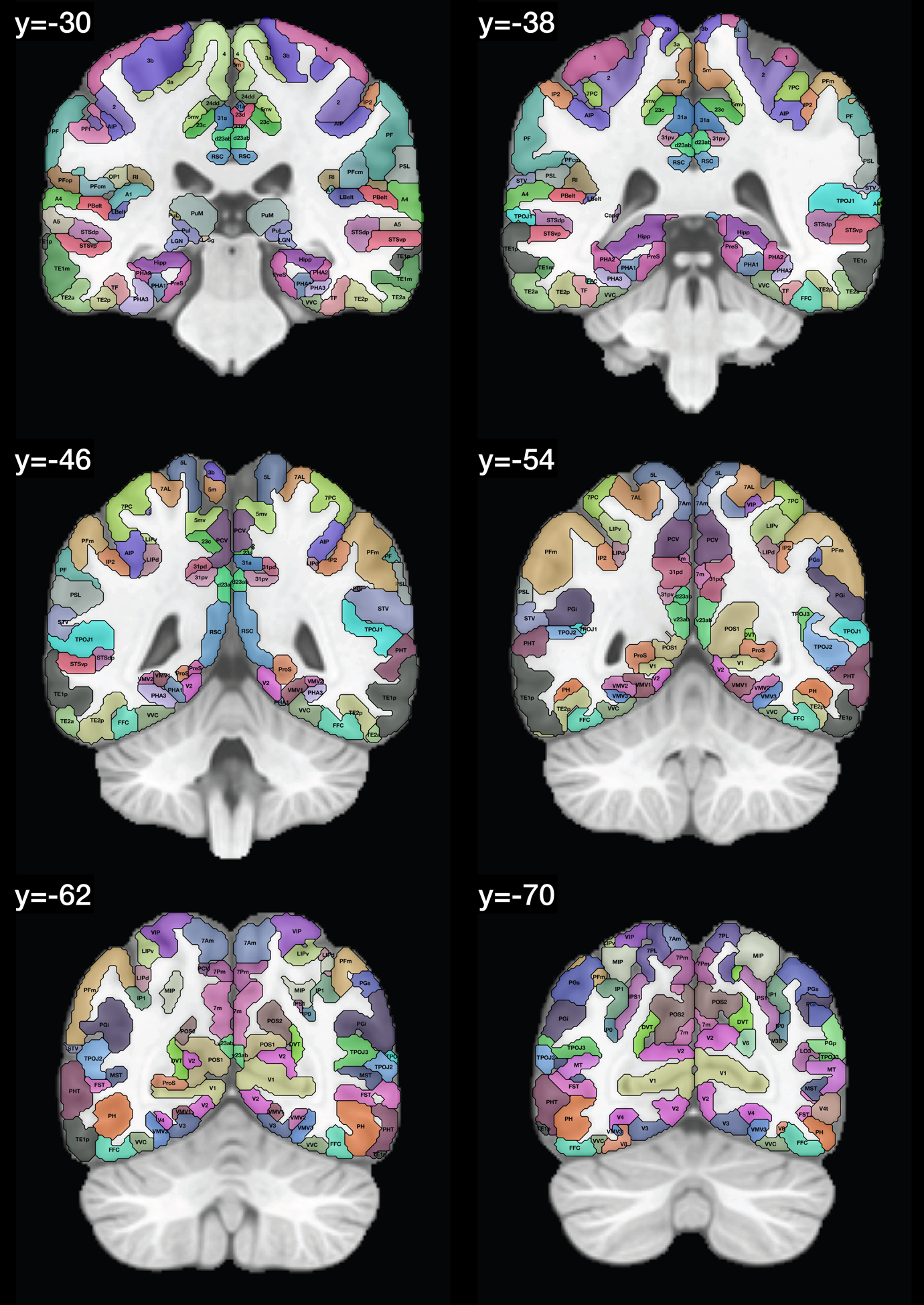


Fig. S1-4. Example coronal slices showing regions defined in the HCPex atlas and added subcortical regions (Huang, et al., 2022). The abbreviations are as in Table S1. The y values for the coronal slices are in MNI coordinates.


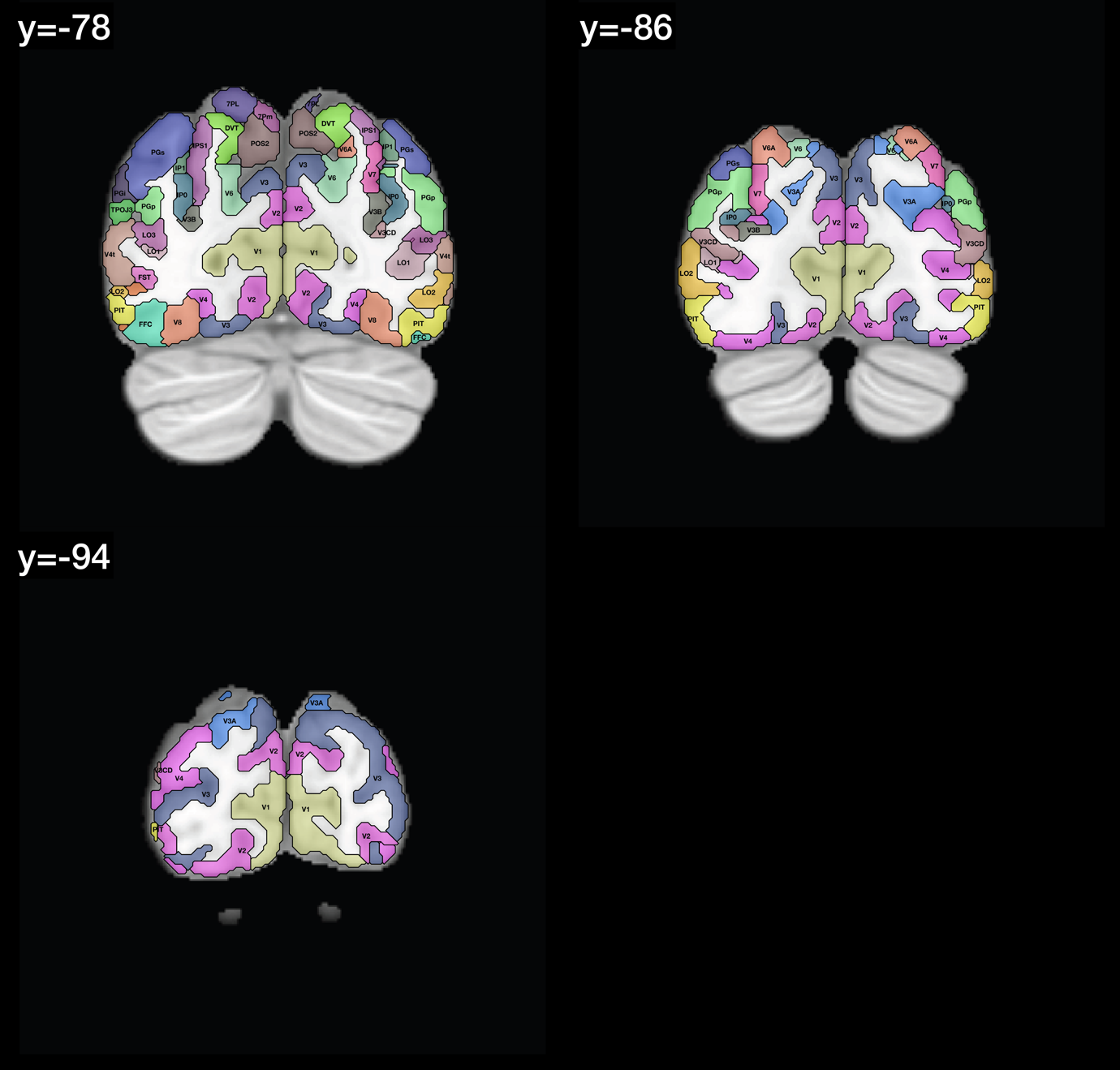


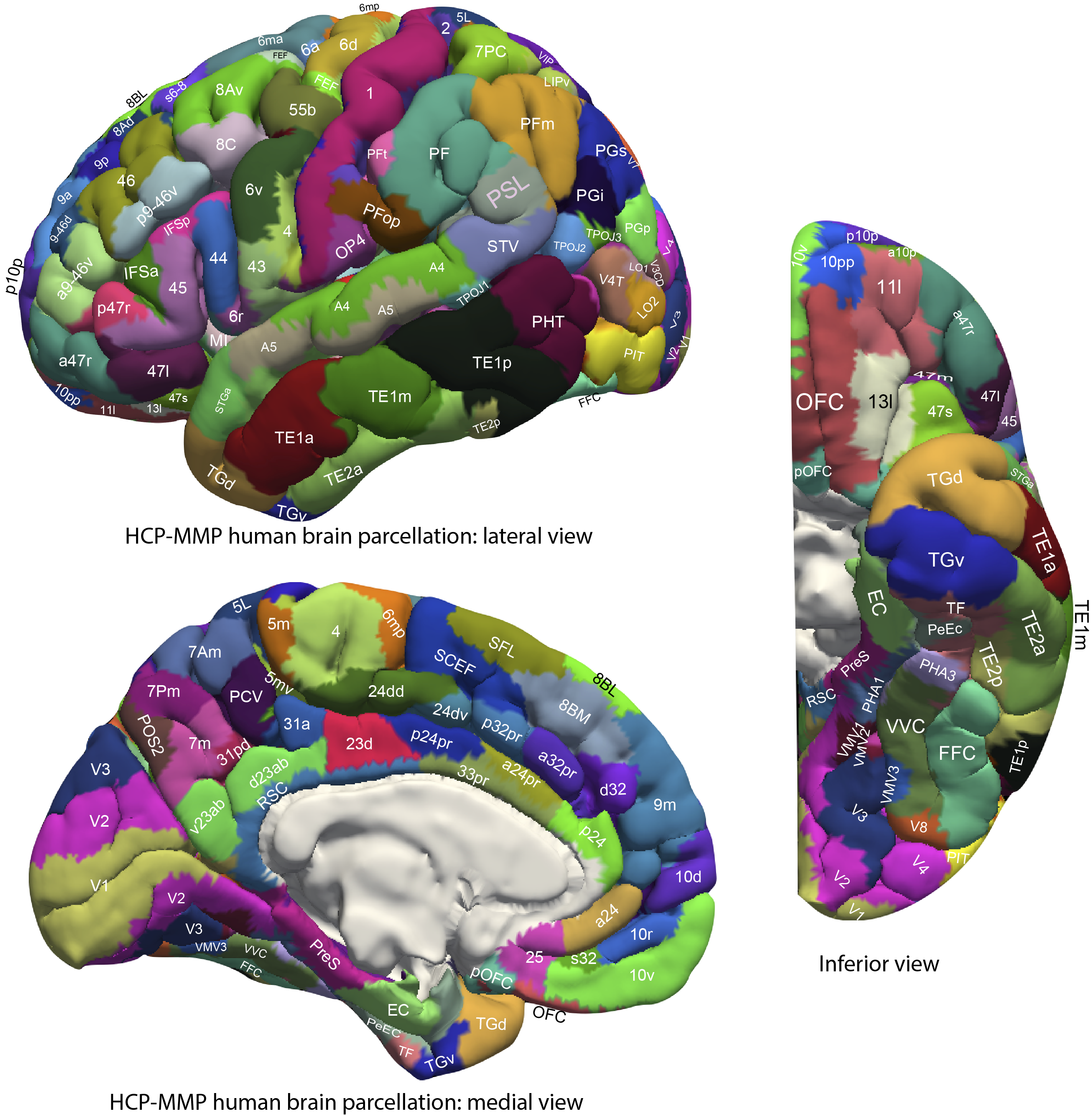


Fig. S1-5. Anatomical regions of the human visual and other cortical regions. Regions are shown as defined in the HCP-MMP atlas (Glasser, et al., 2016), and in its extended version HCPex (Huang, et al., 2022). The regions are shown on images of the human brain without the sulci expanded to show which cortical HCP-MMP regions are normally visible, for comparison with Figs. 6-10. (The ICBM153 MNI T1 image was used to prepare this figure.) Abbreviations are provided in Table S1.

­
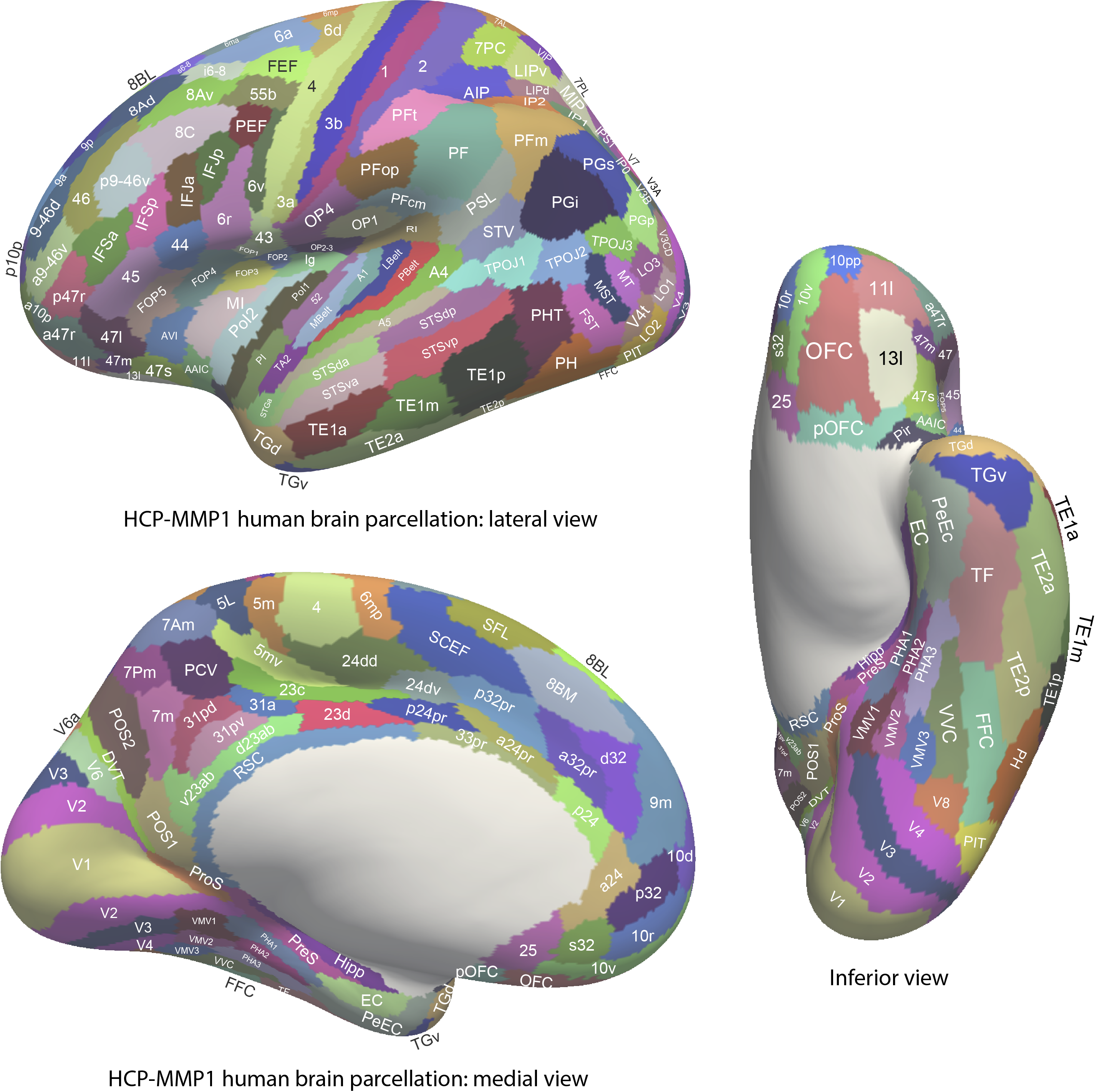


Fig. S1-6. Regions in the Human Connectome Project Multimodal Parcellation atlas (HCP-MMP) (Glasser, et al., 2016) and its extended version HCPex (Huang, et al., 2022) to show the cortical regions. The regions are shown on images of the human brain with the sulci expanded sufficiently to allow the regions within the sulci to be shown. Abbreviations are provided in Table S1. For comparison, a version of this diagram without the sulci expanded is provided in Fig. S1-5. (HCPBrainMaster4bLC.eps)

**Analysis of functional magnetic resonance imaging (fMRI) data**

*The linear Hopf algorithm for effective connectivity*

We used an analytic approach to the calculation of Effective Connectivity described by Deco et al (2023). We confirmed that this analytic algorithm produces similar results to the simulation version of the effective connectivity algorithm described earlier (Rolls, et al., 2022; Rolls, et al., 2023a). A model of whole-brain dynamics is used to represent the local dynamics of each brain area based on a local dynamics expressed by Stuart-Landau oscillators, i.e. by the normal form of a supercritical Hopf bifurcation that is the standard model for examining the shift from noisy to oscillatory dynamics (Kuznetsov, 1998). The intricate connections between Hopf oscillators have been demonstrated to replicate key aspects of brain dynamics seen in electrophysiology (Freyer, et al., 2011; Freyer, et al., 2012), MEG, and other forms of brain network architecture when coupled together (Deco, et al., 2017a) and fMRI (Deco, et al., 2017b; Kringelbach, et al., 2015; Kringelbach and Deco, 2020). More concretely, the whole-brain dynamics with *N* regions can be expressed by coupling the local dynamics of $N$ Stuart-Landau oscillators (i.e. the normal form of a supercritical Hopf bifurcation) interconnected through the connectivity $\boldsymbol{C}$:

$\frac{dz_{j}}{dt}=\left( a_{j}+\text{i}\omega_{j} \right)z_{j}-\left| z_{j} \right|^{2}z_{j}+\sum_{k=1}^{N} C_{jk}\left( z_{k}-z_{j} \right)+\eta_{j}$ (5)

where $z_{j}=x_{j}+\text{i}y_{j}$, $\eta_{j}$ is additive uncorrelated Gaussian noise with variance $\sigma^{2}$ (for all $j)$, $\omega_{j}$ is the intrinsic node frequency, and $a_{j}$ is the node’s bifurcation parameter. Within this model, the intrinsic frequency $\omega_{j}$ of each node is in the 0.04–0.07 Hz band for fMRI and 0.5-2 Hz for MEG analysis. The intrinsic frequencies were estimated from the data, as given by the averaged peak frequency of the narrowband BOLD and MEG signals of each brain region. In equation (5), $\boldsymbol{C}$ is the coupling connectivity matrix. This model can be interpreted as an extension of the Kuramoto model to the case in which both the phase and the amplitude of the oscillators are allowed to vary. In particular, the choice of the coupling function $\left( z_{k}-z_{j} \right)$ promotes phase synchronization between coupled nodes (as can be seen by writing the system in polar coordinates). For $a_{j}>0$, the local dynamics settle into a stable limit cycle, producing self-sustained oscillations with frequency $\frac{\omega_{j}}{2\pi}$. For $a_{j}<0$, the local dynamics present a stable spiral point, producing damped or noisy oscillations in the absence or presence of noise, respectively. The fMRI or MEG signals were modelled by the real part of the state variables, i.e., $x_{j}=\text{Real}\left( z_{j} \right)$.

It was proven (Deco, et al., 2017b) that the best working point for fitting many different neuroimaging based dynamical brain states is given by a working point of each Stuart-Landau oscillator at the brink of the bifurcation, i.e. with $a_{j}$ slightly negative but very near to zero (usually $a_{j}=-0.02$). This is very important, because this allows a linearization of the dynamics that permits an analytical solution of the functional connectivity matrix FC given by the Pearson correlation between the activity of all pairs of brain regions as reflected with BOLD fMRI or MEG. More concretely, we estimate the functional correlations of the whole-brain network using a linear noise approximation (LNA). The dynamical system (5) can be re-written in vector form as:

$\frac{d\boldsymbol{z}}{dt}=\left( \boldsymbol{a}-g\boldsymbol{S}+\text{i}\boldsymbol{\omega} \right)⨀\boldsymbol{z}-\left( \boldsymbol{z}⨀\bar{\boldsymbol{z}} \right)\boldsymbol{z}+G\boldsymbol{Cz}+\boldsymbol{\eta}$ (6)

where $\boldsymbol{z}=\left[ z_{1}, \ldots,z_{N} \right]$, $\boldsymbol{a}=\left[ a_{1}, \ldots,a_{N} \right]$, $\boldsymbol{\omega}=\left[ \omega_{1}, \ldots,\omega_{N} \right]$, $\boldsymbol{S}=\left[ S_{1}, \ldots,S_{N} \right]$ is the vector containing the strength of each node, i.e., $S_{i}=\sum_{j} C_{ij}$, $\boldsymbol{\eta}=\left[ \eta_{1}, \ldots,\eta_{N} \right]$ represents a vector of uncorrelated noise, and $⨀$ is the Hadamard element-wise product. Studying the linear fluctuations around the fixed point $\boldsymbol{z}=\boldsymbol{0}$, which is the solution of $\frac{d\boldsymbol{z}}{dt}=0$, in the linearized system the higher-order terms $\left( \boldsymbol{z}⨀\bar{\boldsymbol{z}} \right)\boldsymbol{z}$ are discarded in equation (6). Using the real and imaginary parts of the state variables, the evolution of the linear fluctuations $\delta\boldsymbol{u}$ follows the following Langevin stochastic linear equation:

$\frac{d}{dt}\delta\boldsymbol{u}=\boldsymbol{J}\delta\boldsymbol{u+\eta}$ (7)

where the 2*N*-dimensional vector $\delta\boldsymbol{u}\boldsymbol{=}\left( \delta\boldsymbol{x}\boldsymbol{,}\delta\boldsymbol{y} \right)\boldsymbol{=}\left( {\delta x}_{1},\ldots,{\delta x}_{N},{\delta y}_{1},\ldots,{\delta y}_{N} \right)$ contains the fluctuations of real and imaginary parts. The $2N\times2N$ matrix $\boldsymbol{J}$ is the Jacobian matrix of the system evaluated at the fixed point. The Jacobian matrix can be written as a block matrix:

$\boldsymbol{J=}\left[ \begin{matrix} \boldsymbol{J}_{\boldsymbol{xx}} & \boldsymbol{J}_{\boldsymbol{xy}} \\ \boldsymbol{J}_{\boldsymbol{yx}} & \boldsymbol{J}_{\boldsymbol{yy}} \end{matrix} \right]$ (8)

where $\boldsymbol{J}_{\boldsymbol{xx}}$, $\boldsymbol{J}_{\boldsymbol{xy}}$, $\boldsymbol{J}_{\boldsymbol{yx}}$, $\boldsymbol{J}_{\boldsymbol{yy}}$ are $N\times N$ matrices given as: $\boldsymbol{J}_{\boldsymbol{xx}}\boldsymbol{=}\boldsymbol{J}_{\boldsymbol{yy}}\text{ = diag}\left( \boldsymbol{a}-g\boldsymbol{S} \right)+g\boldsymbol{C}$ and $\boldsymbol{J}_{\boldsymbol{xy}}\boldsymbol{=}\boldsymbol{-J}_{\boldsymbol{yx}}\text{ = diag}\left( \boldsymbol{\omega} \right)$, where $\text{diag}\text{(}\boldsymbol{v}\text{)}$ is the diagonal matrix whose diagonal is the vector $\boldsymbol{v}$. The linearization is only valid if $\boldsymbol{z}=\boldsymbol{0}$ is a stable solution of the system, i.e., if all eigenvalues of $\boldsymbol{J}$ have negative real part. The motion equation of the covariance matrix $\boldsymbol{K}=\left\langle\delta\boldsymbol{u}{\delta\boldsymbol{u}}^{\boldsymbol{T}} \right\rangle$ (where the superscript $T$ denotes the transpose operator) can be derived by using the linear approximation. This can be done by writing equation (7) as $d\delta\boldsymbol{u}=\boldsymbol{A}\delta\boldsymbol{u}dt\boldsymbol{+}d\boldsymbol{W}$, where $d\boldsymbol{W}$ is an 2*N*-dimensional Wiener process with covariance $\left\langle d\boldsymbol{W}{d\boldsymbol{W}}^{\boldsymbol{T}} \right\rangle=\boldsymbol{Q}_{\boldsymbol{n}}dt$. Using Itô’s stochastic calculus, we get $d\left( \delta\boldsymbol{u}{\delta\boldsymbol{u}}^{\boldsymbol{T}} \right)=d\left( \delta\boldsymbol{u} \right){\delta\boldsymbol{u}}^{\boldsymbol{T}}+\delta\boldsymbol{u}d\left( {\delta\boldsymbol{u}}^{\boldsymbol{T}} \right)+d\left( \delta\boldsymbol{u} \right)d\left( {\delta\boldsymbol{u}}^{\boldsymbol{T}} \right)$. Noting that $\left\langle\delta\boldsymbol{u}{d\boldsymbol{W}}^{\boldsymbol{T}} \right\rangle=0$, tacking the expectations and keeping terms in the first order of the differential $dt$, we obtain:

$\frac{d\boldsymbol{K}}{dt}=\boldsymbol{J}\boldsymbol{K+K}\boldsymbol{J}^{\boldsymbol{T}}\boldsymbol{+}\boldsymbol{Q}_{\boldsymbol{n}}$ (9)

where the covariance matrix of the noise $\boldsymbol{Q}_{\boldsymbol{n}}$ is diagonal for uncorrelated noise. Hence, the stationary covariances (for which $\frac{d\boldsymbol{K}}{dt}=0$) can be obtained by solving the following algebraic equation:

$\boldsymbol{J K+K}\boldsymbol{J}^{\boldsymbol{T}}\boldsymbol{+}\boldsymbol{Q}_{\boldsymbol{n}}\boldsymbol{=0}$ (10)

Equation (10) is a Lyapunov equation that can be solved using the eigen-decomposition of the Jacobian matrix (Deco, et al., 2014). To solve it numerically, we used the Matlab function *lyap.m*. We obtained the simulated functional connectivity $\boldsymbol{FC}^{model}$ from the first N rows and columns of the covariance $\boldsymbol{K}$ corresponding to the real part of the dynamics which is precisely used to model the BOLD fMRI or MEG signal.

In order to fit the model to the empirical data (BOLD fMRI or MEG signal of each subject in each condition, i.e. brain state), we ran a pseudo-gradient procedure aiming to optimize the coupling connectivity matrix $\boldsymbol{C}$ . We optimized this generative effective coupling matrix between brain regions by comparing the output of the model with the empirical measures of the correlation matrix ($\boldsymbol{FC}^{empirical}$) and of the normalized $\tau$ time-shifted covariances ($\boldsymbol{F}\boldsymbol{S}^{empirical}\left( \tau\right)$) given by the shifted covariance matrix $\boldsymbol{KS}^{empirical}\left( \tau\right)$ dividing each pair ($i$, $j$) by $\sqrt{{KS}_{ii}^{empirical}{\left( 0 \right)KS}_{jj}^{empirical}\left( 0 \right)}$. Note that the shifted covariance breaks the symmetry of the coupling and thus improves the level of fitting and in particular enables measurement of the strength of the effective connectivity in both directions between each pair of nodes (cf. Gilson, et al., 2016). Using a heuristic gradient algorithm, we proceed to update the $\boldsymbol{C}$ such that the fit is optimised. More specifically, the updating uses the following form:

$$C_{ij}=C_{ij}+\varepsilon_{1}\left( {FC}_{ij}^{empirical}-{FC}_{ij}^{model} \right)$$

$+\varepsilon_{2}\left( {FS}_{ij}^{empirical}\left( \tau\right)-{FS}_{ij}^{model}\left( \tau\right) \right)$ (11)

where ${FS}_{ij}^{model}\left( \tau\right)$ is given by the first N rows and columns of the simulated $\tau$ time-shifted covariances $\boldsymbol{KS}^{model}\left( \tau\right)$ normalized by dividing each pair ($i$, $j$) by $\sqrt{{KS}_{ii}^{model}{\left( 0 \right)KS}_{jj}^{model}\left( 0 \right)}$, with $\boldsymbol{KS}^{model}\left( \tau\right)$ the shifted simulated covariance matrix computed as follows:

$\boldsymbol{KS}^{model}\left( \tau\right)=\exp\left( \tau\boldsymbol{J} \right) \boldsymbol{K}$ (12)

Note that $\boldsymbol{KS}^{model}\left( 0 \right)\boldsymbol{=K}$. The model was run repeatedly with the updated $\boldsymbol{C}$ until the fit converged towards a stable value.

We initialised $\boldsymbol{C}$ in the same way as with the simulation version of the Hopf effective connectivity algorithm (Rolls, et al., 2022; Rolls, et al., 2023a). We used $\varepsilon_{1}=0.0004$ for the Functional Connectivity terms in Equation 11, and $\varepsilon_{2}=0.0001$ for the time delayed covariance term, and found that higher values could cause the algorithm to not converge correctly. The same applies to the simulation version of the algorithm. For the results described here, we computed the mean of *FC*^empirical^ and of *FS*^empirical^ across subjects, and used these as the input to the Hopf algorithm. With the 23 participants in the fMRI analyses described here, we were able to validate the results by splitting the data into two halves, and found that the 360x360 effective connectivity matrices computed with different participants were correlated with r=0.8. As described elsewhere in effective connectivity investigations with magnetoencephalography (Rolls, et al., 2023b; Rolls, et al., 2024), the direction of the effective connectivity measured with fMRI needs to be and was reversed in this and in previous investigations with this algorithm in order to provide the forward direction up through sensory hierarchies. This is probably related to the slow timecourse with fMRI, as set out elsewhere (Rolls, et al., 2023b; Rolls, et al., 2024), and is not necessary when the same algorithm is used with magnetoencephalography (Rolls, et al., 2023b; Rolls, et al., 2024).

References

Deco, G., Ponce-Alvarez, A., Hagmann, P., Romani, G.L., Mantini, D., Corbetta, M. (2014) How local excitation-inhibition ratio impacts the whole brain dynamics. J. Neurosci., 34:7886-98.

Deco, G., Cabral, J., Woolrich, M.W., Stevner, A.B.A., van Hartevelt, T.J., Kringelbach, M.L. (2017a) Single or multiple frequency generators in on-going brain activity: A mechanistic whole-brain model of empirical MEG data. Neuroimage, 152:538-550.

Deco, G., Kringelbach, M.L., Jirsa, V.K., Ritter, P. (2017b) The dynamics of resting fluctuations in the brain: metastability and its dynamical cortical core. Sci. Rep., 7:3095.

Deco, G., Lynn, C.W., Sanz Perl, Y., Kringelbach, M.L. (2023) Violations of the fluctuation-dissipation theorem reveal distinct nonequilibrium dynamics of brain states. Phys Rev E, 108:064410.

Freyer, F., Roberts, J.A., Becker, R., Robinson, P.A., Ritter, P., Breakspear, M. (2011) Biophysical mechanisms of multistability in resting-state cortical rhythms. J. Neurosci., 31:6353-61.

Freyer, F., Roberts, J.A., Ritter, P., Breakspear, M. (2012) A canonical model of multistability and scale-invariance in biological systems. PLoS Comput. Biol., 8:e1002634.

Gilson, M., Moreno-Bote, R., Ponce-Alvarez, A., Ritter, P., Deco, G. (2016) Estimation of directed effective connectivity from fMRI functional connectivity hints at asymmetries in the cortical connectome. PLoS Comput. Biol., 12:e1004762.

Glasser, M.F., Coalson, T.S., Robinson, E.C., Hacker, C.D., Harwell, J., Yacoub, E., Ugurbil, K., Andersson, J., Beckmann, C.F., Jenkinson, M., Smith, S.M., Van Essen, D.C. (2016) A multi-modal parcellation of human cerebral cortex. Nature, 536:171-8.

Huang, C.C., Rolls, E.T., Feng, J., Lin, C.P. (2022) An extended Human Connectome Project multimodal parcellation atlas of the human cortex and subcortical areas. Brain Struct Funct, 227:763-778.

Kringelbach, M.L., McIntosh, A.R., Ritter, P., Jirsa, V.K., Deco, G. (2015) The rediscovery of slowness: exploring the timing of cognition. Trends Cogn. Sci., 19:616-628.

Kringelbach, M.L., Deco, G. (2020) Brain states and transitions: insights from computational neuroscience. Cell Rep, 32:108128.

Rolls, E.T., Deco, G., Huang, C.C., Feng, J. (2022) The effective connectivity of the human hippocampal memory system. Cereb. Cortex, 32:3706-3725.

Rolls, E.T., Deco, G., Huang, C.-C., Feng, J. (2023a) Multiple cortical visual streams in humans. Cereb. Cortex, 33:3319-3349.

Rolls, E.T., Deco, G., Zhang, Y., Feng, J. (2023b) Hierarchical organization of the human ventral visual streams revealed with magnetoencephalography. Cereb. Cortex, 33:10686-10701.

Rolls, E.T., Yan, X., Deco, G., Zhang, Y., Jousmaki, V., Feng, J. (2024) A ventromedial visual cortical 'Where' stream to the human hippocampus for spatial scenes revealed with magnetoencephalography. Communications Biology, 7:1047.
